# Analogy Powered by Prediction and Structural Invariants: Computationally Led Discovery of a Mesoporous Hydrogen-Bonded Organic Cage Crystal

**DOI:** 10.1021/jacs.2c02653

**Published:** 2022-05-29

**Authors:** Qiang Zhu, Jay Johal, Daniel E. Widdowson, Zhongfu Pang, Boyu Li, Christopher M. Kane, Vitaliy Kurlin, Graeme M. Day, Marc A. Little, Andrew I. Cooper

**Affiliations:** † Materials Innovation Factory and Department of Chemistry, University of Liverpool, Liverpool L7 3NY, U.K.; ‡ Leverhulme Research Centre for Functional Materials Design, University of Liverpool, Liverpool L7 3NY, U.K.; § Computational Systems Chemistry, School of Chemistry, University of Southampton, Southampton SO17 1BJ, U.K.; ∥ Computer Science, University of Liverpool, Liverpool L69 3BX, U.K.

## Abstract

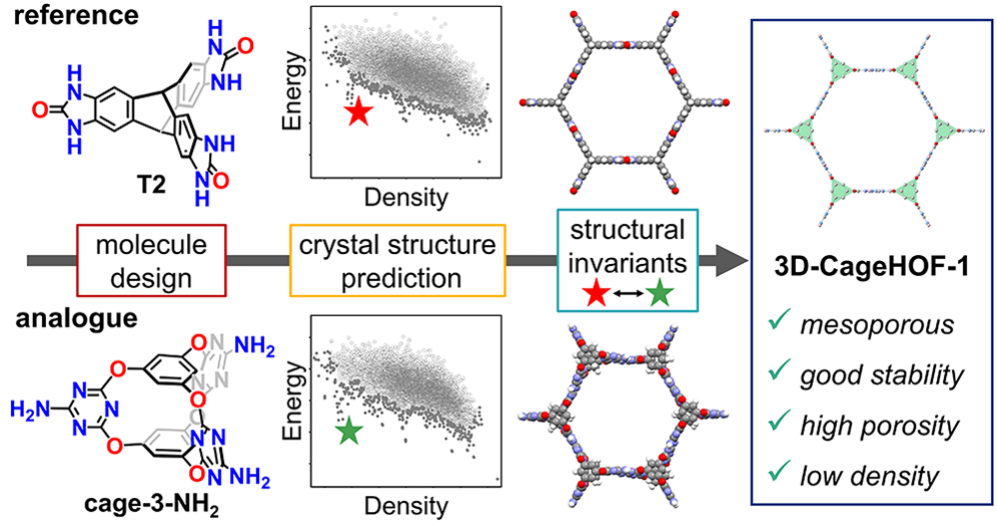

Mesoporous molecular crystals have potential applications in separation and catalysis, but they are rare and hard to design because many weak interactions compete during crystallization, and most molecules have an energetic preference for close packing. Here, we combine crystal structure prediction (CSP) with structural invariants to continuously qualify the similarity between predicted crystal structures for related molecules. This allows isomorphous substitution strategies, which can be unreliable for molecular crystals, to be augmented by *a priori* prediction, thus leveraging the power of both approaches. We used this combined approach to discover a rare example of a low-density (0.54 g cm^–3^) mesoporous hydrogen-bonded framework (HOF), **3D-CageHOF-1**. This structure comprises an organic cage (**Cage-3-NH**_**2**_) that was predicted to form kinetically trapped, low-density polymorphs *via* CSP. Pointwise distance distribution structural invariants revealed five predicted forms of **Cage-3-NH**_**2**_ that are analogous to experimentally realized porous crystals of a chemically different but geometrically similar molecule, **T2**. More broadly, this approach overcomes the difficulties in comparing predicted molecular crystals with varying lattice parameters, thus allowing for the systematic comparison of energy–structure landscapes for chemically dissimilar molecules.

## Introduction

The development of reliable methods for crystal structure prediction (CSP)^1,2^ provides a powerful tool for the *ab initio* discovery of porous molecular crystals^3,4^ and other functional organic solids. The lowest energy structures resulting from a computational search are assumed to be the most likely to be observed experimentally, and the probability of observing a particular structure can be related to its energy.^5^ In the context of functional material discovery, CSP allows an assessment of a molecule’s tendency to form crystal structures with an arrangement of molecules that provides the desired property, therefore guiding experimental workflows. Such guides can be expressed graphically through energy–structure–function maps that show the relationship between lattice energy and computed function for a molecule.^3,6^

However, predicting whether a molecule can form a stable porous crystal poses a challenge because such crystal packings often correspond to kinetically trapped, high-energy structures.^3,7−9^ One approach is to apply *a priori* CSP coupled with computational methods for assessing solvent templating^8^ and kinetic stability,^10^ followed by the experimental screening of crystallization conditions for the molecules that are computed to have likely porous structures. An alternative, much more established approach makes use of analogy, substituting the molecule in a known porous structure with a related molecule that is expected, in principle, to be capable of adopting the same crystal packing arrangement. Such “isomorphous substitution” strategies are the basis for the reticular chemistry methods that have proved highly successful for designing porous bonded frameworks, such as metal–organic frameworks (MOFs)^11^ and covalent organic frameworks (COFs).^12,13^ By contrast, the crystallization of organic molecules is dependent on weaker and less directional intermolecular interactions, which makes crystallization outcomes much more difficult to predict, even for structurally similar molecular building blocks. Hence, there is an opportunity to augment isomorphous substitution methods for organic molecules beyond simple analogy by applying CSP strategies.

Porous hydrogen-bonded frameworks (HOFs) have potential applications, such as gas storage, molecular separations, catalysis, sensing, solid electrolytes, and enzyme encapsulation.^14−17^ Typically, HOFs are designed by appending hydrogen-bonding units to organic scaffolds to control their assembly.^14−16^ An advantage of HOFs is that they are often highly crystalline. However, most hydrogen-bonding molecules have an energetic preference for close packing, and HOFs that do crystallize with low framework densities are frequently unstable to desolvation. More recently, some HOFs with excellent chemical and physical stabilities have been discovered,^9,18−21^ although the isostructural series of porous HOFs remain much rarer^14−16^ than isoreticular MOFs^11,22^ and COFs^12,13,23^ for the reasons outlined above. A related challenge for HOFs with large mesopores is the tendency to form interpenetrated structures,^7,24−32^ which have lower porosity levels.

A challenge for the coupled use of CSP with isomorphous substitution strategies is to define whether two phases of related but structurally dissimilar molecules are analogous or isostructural. Such comparisons are often simple to make by eye—for example, to say that two dissimilar molecules both pack as hexagonal, hydrogen-bonded nets—but it is harder to define these similarities in a formal, quantitative way. Common methods for comparing crystal structure similarity, such as root-mean-square deviation (RMSD) of atomic positions, break down when applied to dissimilar molecules. It is therefore challenging to provide a formal metric to define when the crystal packing of molecule A is “like” the crystal packing of dissimilar molecule B. This is a general challenge in supramolecular chemistry that goes beyond comparing crystal packings on CSP landscapes.

**Figure 1:**
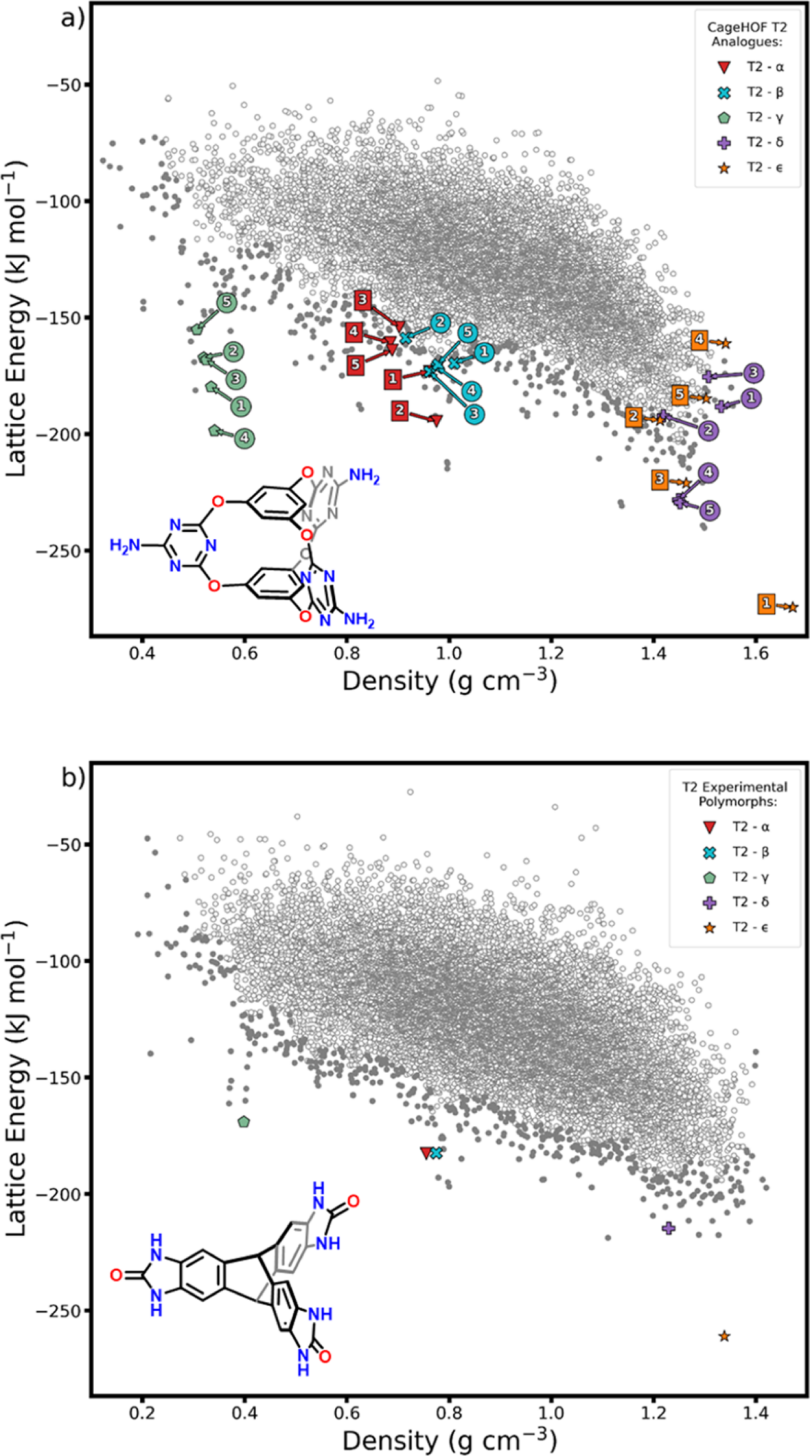
Energy–density distributions of the CSP structures of (a) **Cage-3-NH**_**2**_ and (b) **T2**. In both cases, the “leading edge” structures are shown as filled gray points to highlight those that are most likely to be found by experiment. The best matches to the five experimentally observed polymorphs of **T2** are indicated on the **T2** landscape, based on comparisons made using the COMPACK algorithm. On the **Cage-3-NH**_**2**_ landscape, we highlight the five closest analogues of each **T2** polymorph, based on isometry invariants (discussed below). These comparisons were restricted to the leading edge of the **Cage-3-NH**_**2**_ energy–density distribution, and their crystal structures are shown in Figure 2.

In this study, we use structural invariants—that is, geometry-based crystal descriptors that can continuously assess similarity—to compare predicted crystal structures of a trigonal cage molecule, **Cage-3-NH**_**2**_ (Figure 1a),^33^ with five known polymorphs of a trigonal triptycene benzimidazolone molecule (**T2**, Figure 1b).^3,34^ We chose **T2** because of its rich polymorphic behavior and its geometric similarity with **Cage-3-NH**_**2**_. Specifically, we were interested in searching for a structural analogue of the lowest density (0.42 g cm^–3^), highly porous polymorph, **T2**-γ, which has a Brunauer–Emmett–Teller (BET) surface area of 3425 m^2^ g^–1^.^3^ Initially, we used CSP to predict the energy landscape of **Cage-3-NH**_**2**_. We then used a new structural invariant^35,36^ to continuously quantify the similarity between five polymorphs of **T2**(3,34) and the predicted crystal structures of **Cage-3-NH**_**2**_. This new approach analyzes CSP results by comparison with structural analogues, and this helped us to identify a rare example of a mesoporous HOF (**3D-CageHOF-1**) that was kinetically trapped on the **Cage-3-NH**_**2**_ energy landscape. This predicted HOF structure was subsequently prepared in the laboratory and has a low density, with 2.3 nm sized one-dimensional (1D) pores and an experimental BET surface area of 1750 m^2^ g^–1^. Our design strategy mimics the isoreticular approaches developed for MOFs^11,22^ and COFs^13,23^ by identifying chemically different building blocks that can form kinetically trapped materials with related porous structures.

Organic cage molecules with various topologies and cavity sizes are now synthetically accessible,^37,38^ and their one-pot self-assembly can be screened using computational methods.^39^ Here, we demonstrate a new strategy to predict the crystal structures of cage molecules and to design stable, porous HOFs, complementing isoreticular approaches used to design cage-based MOFs^40,41^ and COFs.^42−44^ Thinking beyond porous solids, the comparison of related CSP landscapes offers a formal method for the *a priori* design of functional materials by analogy, using known functional solids as the starting point.

## Results and Discussion

Solution processible organic cages are versatile three-dimensional (3D) building blocks for constructing extended materials^45,46^ and close-packed porous crystals.^4^ Appending organic cages with hydrogen-bonding units to modulate their 3D packing has only been investigated recently: the first cage-based HOF was reported by Han *et al.*,^33^ who found that the triangular prism-shaped cage, **Cage-3-NH**_**2**_ (Figure 1a), formed HOF-19, which has a two-dimensional (2D) ladder-like structure (Figure S13). By extension, we were interested in exploring the crystallization behavior of **Cage-3-NH**_**2**_ to determine whether it could form other porous crystal structures, assisted by computational prediction.

### CSP Calculations

The CSP energy–density landscape for **Cage-3-NH**_**2**_ is shown in Figure 1a. These initial calculations used quasi-random structure generation,^47^ a rigid-molecule approximation, and an empirically parametrized intermolecular force field with atom-centered electrostatic multipoles^48^ (see the Supporting Information, Section S1, Figures S1–S5, and Tables S1–S3).

As usual, density is correlated with energy: the lowest energy structures are among the densest. However, multiple spikes are apparent on the leading edge of the landscape. These spikes correspond to unusually low energy structures being predicted in several narrow density ranges. The presence of these spikes on CSP energy–density plots has, in several cases, anticipated the experimental discovery of kinetically trapped porous molecular crystals.^3,7,9^ Recent computational work has shown that these spikes correspond to deep energy basins, separated from dense crystal packings by high energy barriers.^10^

**Figure 2:**
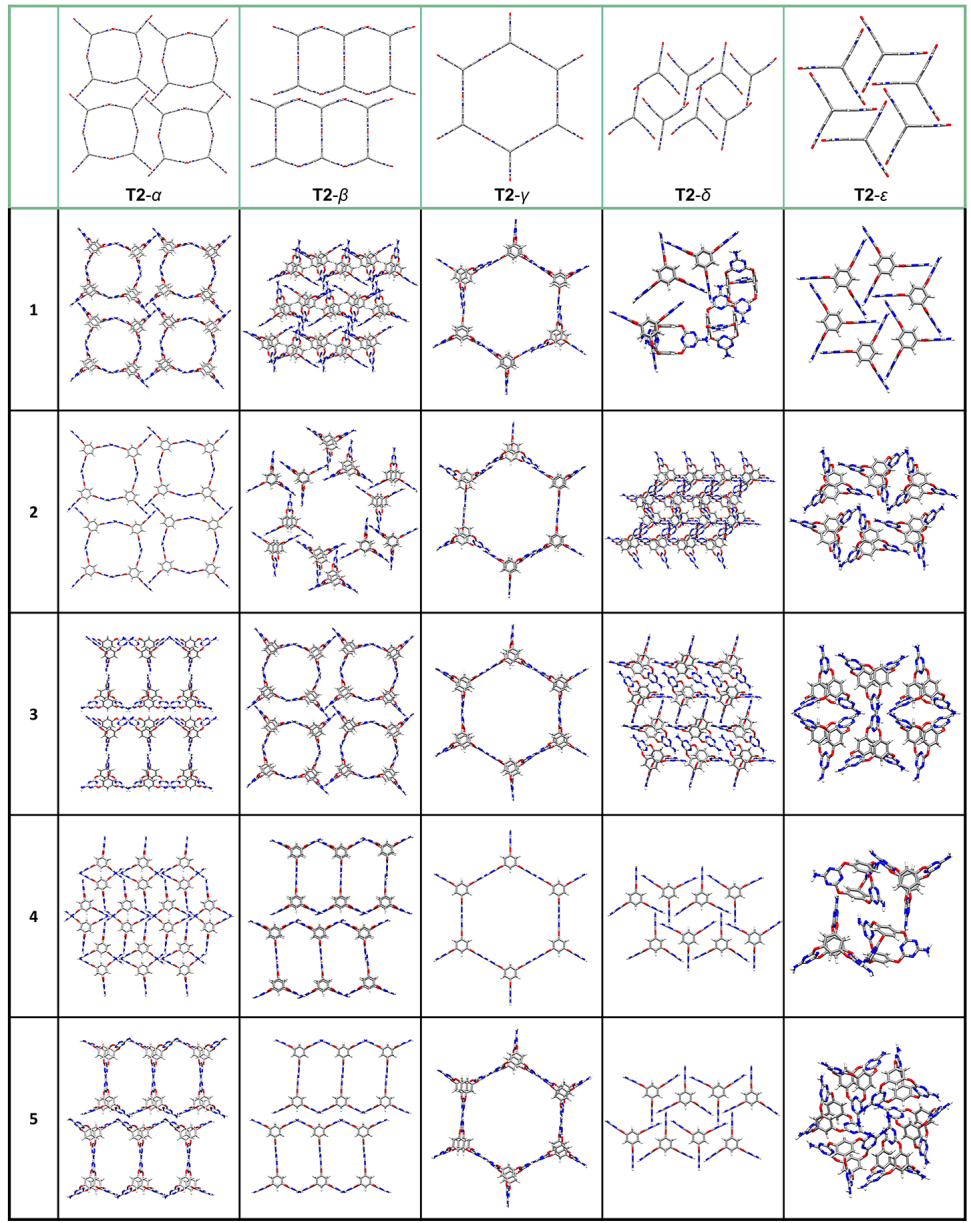
Crystal packing diagrams of predicted **Cage-3-NH**_**2**_ structures from the pre-DFTB-optimized CSP data set that were identified *via* structural invariants as the nearest neighbors to the **T2**-α, **T2**-β, **T2**-γ, **T2**-δ, and **T2**-ε polymorphs (from **1** to **5**, **1** being the nearest neighbor).

The close-packed global energy minimum predicted structure of **Cage-3-NH**_**2**_ has a density of 1.67 g cm^–3^, but prominent spikes on the CSP landscape are apparent at densities close to 1.34, 1.00, 0.83, and 0.54 g cm^–3^. The overall structure of the crystal energy landscape, including the low-density spikes, is strongly reminiscent of that reported for **T2** (Figure 1b),^34^ which has the same trigonal *D*_3*h*_ symmetry as **Cage-3-NH**_**2**_ and an analogous arrangement of hydrogen bond donors and acceptors at the ends of the three “arms” of the molecule. The spike at 1 g cm^–3^ on the **Cage-3-NH**_**2**_ energy–density plot was found to contain predicted structures with high similarity to the HOF-19 structure reported by Han *et al.*,^33^ which is closely related to the β polymorph of **T2** (Figure 2, top row).^3^ The visualization of the predicted structures along the leading edge of the CSP landscape of **Cage-3-NH**_**2**_ finds structures with similar crystal packing to those predicted for **T2** (Figures S4 and S5 and Table S3).^3^

When comparing CSP structures to the reported experimental crystal structure of HOF-19, we noted distortion of the molecular geometry away from the ideal *D*_3*h*_ geometry. Therefore, to improve the quality of the most important CSP structures, we re-optimized the 386 structures on the leading edge of the energy–density distribution using density functional theory-based tight binding (DFTB), which allowed for molecular flexibility within each crystal structure (Table S2, Figure S3). After re-optimization, one of the CSP structures provides a good match to the HOF-19 structure reported by Han *et al.*(33) For comparison, the **T2** leading edge structures were also re-optimized with DFTB. Energy–density distributions after re-optimization are shown in Figures S1 and S2 (Supporting Information).

### Structural Invariants

We used a new isometry invariant to continuously quantify the similarity of the two crystal energy landscapes for **T2** and **Cage-3-NH**_**2**_. Our aim was to determine whether structural invariants could identify analogous structures on the **Cage-3-NH**_**2**_ landscape that matched the four reported **T2** polymorphs, **T2**-α, **T2**-β, **T2**-γ, and **T2**-δ, plus a new densely packed **T2** polymorph, reported here for the first time, **T2**-ε, which was grown by sublimation at *ca.* 800–850 °C using a tube furnace under reduced pressure (∼3.5 × 10^–2^ mbar) (see the Supporting Information, Section S3.1 for refinement and structural details). We note that such analogous crystal structures cannot be identified routinely in molecular crystal data sets because searches using unit cell dimensions or crystal packing similarities otherwise fail; for example, while both **Cage-3-NH_2_** and **T2** are trigonal, **Cage-3-NH**_**2**_ has a different size and a different aspect ratio.

The structural invariant used here was the pointwise distance distribution (PDD) defined for a periodic set of points. Here, we used the center of mass of the molecules as the periodic points (see the Supporting Information, Section S2). The PDD of a periodic set (*S*) is obtained from the matrix *m* × *k* in which each row consists of ordered distances from a point (*p*) in a unit cell of *S* to a number (*k*) of its nearest neighbors in *S*. In the *m* × *k* matrix, the distance rows are lexicographically ordered. If any rows are identical, they collapse into a single row in the matrix, and a weighting is applied (Figure S8). The resulting PDD(*S*; *k*) is a weighted distribution of distance rows, independent of a unit cell,^36^ invariant under isometry (composition of translations, rotations, and reflections). Here, we use the 100 nearest neighbors as the *k* value. The earth mover’s distance was previously used to compare crystal compositions^49^ and is now adapted to a continuous metric between PDDs (Tables S4 and S5), which is easier to compute than between complete isoset invariants.^50^ The PDD is more robust and quicker to compute than past invariants,^51−53^ which allowed it to be used to distinguish all the periodic crystals in the Cambridge Structural Database.^36^

In Figure 2, we highlight the five predicted **Cage-3-NH**_**2**_ crystal structures with the highest similarity from the leading edge of the CSP energy–density distribution, as measured by PDD invariants, to each of the five known polymorphs of **T2**. Using these geometrical comparisons, we find a strong correspondence between the two energy landscapes (Figure 1). The **Cage-3-NH**_**2**_ global energy minimum has a packing that is analogous to the close-packed global energy minimum **T2**-ε polymorph, and this is identified as the nearest neighbor (**1**, Figure 2) using the PDD invariant. In addition, the PDD invariant identified that the leading edge of the **Cage-3-NH**_**2**_ landscape is populated by structures that are analogous to the four known porous **T2** polymorphs: **T2**-α (structures **1–2**, Figure 2), **T2**-β (**4–5**, Figure 2), **T2**-γ (**1–5**, Figure 2), and **T2**-δ (**4–5**, Figure 2), albeit with more pronounced differences in crystal packing noted for **T2**-δ. There are 386 predicted structures on the leading edge of the **Cage-3-NH**_**2**_ energy landscape, and the PDD invariants locate packings that are isostructural to each of the five known **T2** polymorphs within the top five closest **Cage-3-NH**_**2**_ neighbors (Figure 2). This illustrates the power of this computationally inexpensive metric to automate structure comparisons. In the future, this method should allow the cross comparison of entire structure–energy landscapes.

The prediction of **T2** analogous structures on the CSP landscape of **Cage-3-NH**_**2**_ prompted us to pursue these structures experimentally. We were particularly interested in finding conditions that lead to the low-energy structure in the spike at 0.54 g cm^–3^, which was identified as the nearest neighbor of **T2**-γ, and which is a non-interpenetrated 3D HOF with a 2.2 nm sized pore (Figure 2).

### Crystallization Studies

**Figure 3:**
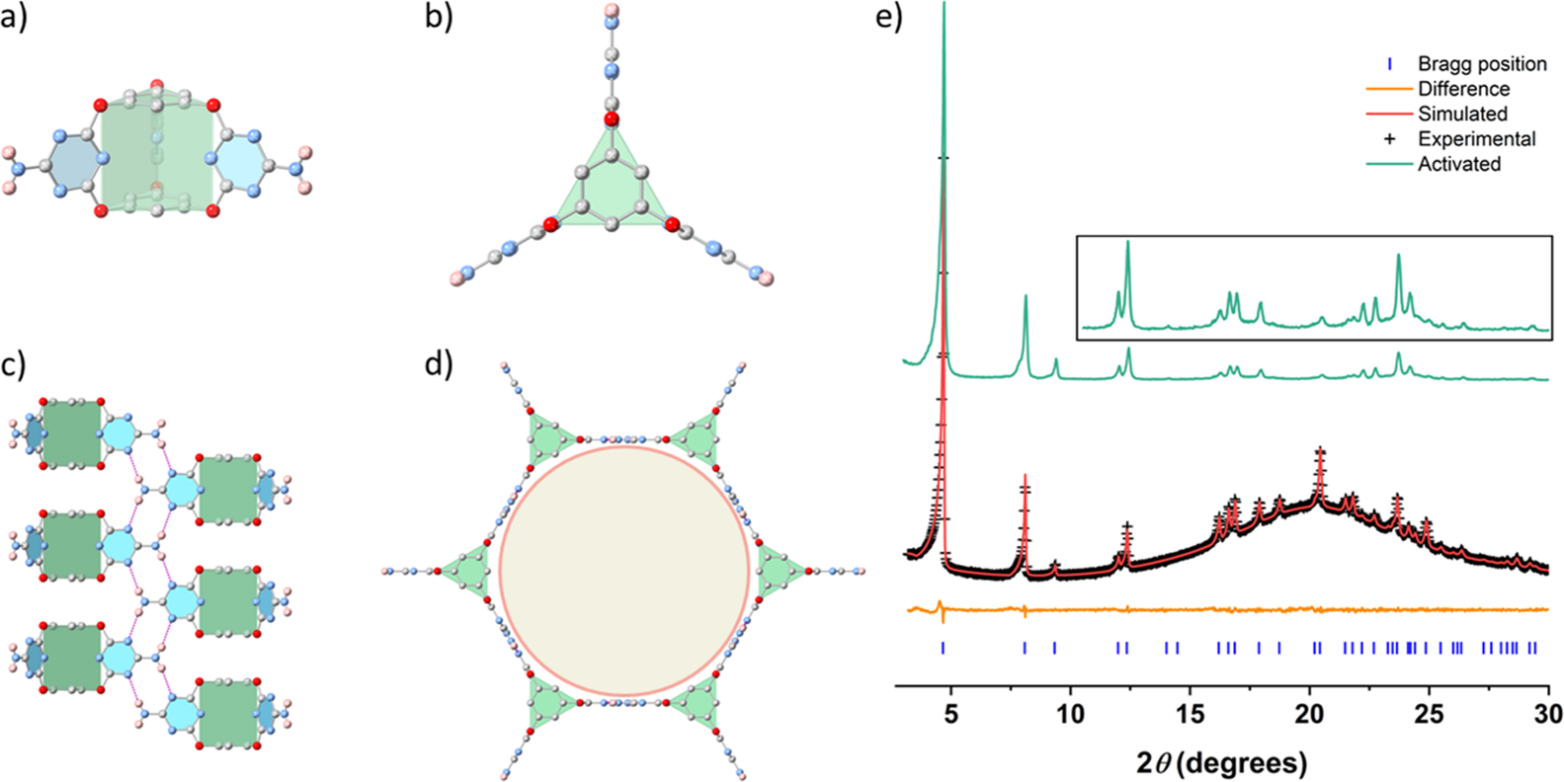
Crystal structures and stability of **3D-CageHOF-1**. Front view (a) and top view (b) of **Cage-3-NH**_**2**_ in the sc-XRD structure of **3D-CageHOF-1**; front view (c) and top view (d) of **3D-CageHOF-1**; single-crystal atom colors: C, gray; N, blue; O, red; and H, pink. H atoms on the phenyl rings are omitted for clarity. (e) PXRD pattern fitting of solvated **3D-CageHOF-1** with Pawley refinement (Cu-Kα) and the activated PXRD pattern of **3D-CageHOF-1** (green). In the insert, the peak intensities are multiplied by 10 from 2*θ* = 10.

CSP does not tell us how to access a particular predicted structure. Computational methods have been used to predict solvent effects on the crystallite size dependence of polymorph stability^54^ and to screen solvent stabilization effects on CSP predicted crystal structures.^3,8^ Although methods such as these could be used to guide the choice of crystallization conditions, such computational methods are expensive when applied to large CSP structure sets and were not used in this study. Instead, we studied the crystallization behavior of **Cage-3-NH**_**2**_ experimentally (see the Supporting Information, Section S5). Han *et al.*(33) reported that crystallizing **Cage-3-NH**_**2**_ from formic acid afforded HOF-19^33^ (Figure S13). Here, we found that slowly diffusing diethyl ether into a solution of **Cage-3-NH**_**2**_ dissolved in formic acid afforded small needle-shaped crystals (Figure S14). Aniline (1 M equiv per **Cage-3-NH**_**2**_) was used as a modulator to slow down the crystallization process and enable us to grow large enough crystals for single-crystal X-ray diffraction (sc-XRD) of what appeared to be the same material by powder XRD (PXRD, Figures S14 and S15). sc-XRD analysis revealed that the needle-shaped crystals had *P*6_3_/*mmc* symmetry in which the **Cage-3-NH**_**2**_ cage has a near-perfect triangular prismatic topology, and the −NH_2_ groups hydrogen bond to triazine N atoms in neighboring cages. In total, each **Cage-3-NH**_**2**_ hydrogen bonds to six adjacent neighbors *via* this motif to form a 3D hydrogen-bonded network (**3D-CageHOF-1**, Figure 3, see the Supporting Information, Section S3.1 for refinement and structural details). The networks have a honeycomb shape with 2.2 nm wide hexagonal 1D channels directed along the crystallographic *c*-axis. The hydrogen bond distance was calculated to be 2.98 Å, and the shortest distance on the *c*-axis between adjacent cages was 3.44 Å, indicating that there are additional π–π stacking interactions between the cages. Remarkably, the accessible void volume of **3D-CageHOF-1** is 72.5% of the unit cell volume, which is among the highest values reported for HOFs (Table S6).^14^

The CSP structure that was the target for experimental searches showed a strong match with the experimental structure, with 30 out of 30 molecules in common and an RMSD of atomic positions of 0.267 Å using CSD Mercury software^55^ (Tables S1 and S2). As such, CSP led us to a new mesoporous polymorph of **Cage-3-NH**_**2**_ that has a much lower framework density than the previously reported HOF-19.

### Characterization of **3D-CageHOF-1**

HOFs with mesopores remain rare but are desirable as highly porous molecular crystals.^20,29,56^ To measure the experimental porosity of **3D-CageHOF-1**, we scaled up the crystallization containing 1 M equiv of aniline per **Cage-3-NH**_**2**_, and characterized the bulk material by PXRD (Figures S16). A Pawley refinement of the solvated material matched well with the simulated PXRD pattern of **3D-CageHOF-1** and indicated the sample was phase pure (Figure 3e, *P*6_3_/*mmc*, *a* = *b* = 21.87 Å, *c* = 8.01 Å, *V* = 3316 Å^3^, *R*_wp_ = 1.41%, *R*_p_ = 1.06%, χ^2^ = 1.22). After confirming the phase purity of the sample, we used supercritical CO_2_ (scCO_2_) to activate the crystal pores after exchanging the crystallization with acetone. PXRD was again used to confirm the phase purity of **3D-CageHOF-1** after scCO_2_ activation (Figures S17–S19). After degassing the scCO_2_ activated **3D-CageHOF-1** under a dynamic vacuum at 25 °C for 15 h, we confirmed that crystallization solvents were removed from the pores by thermogravimetric analysis (TGA) (Figure S20) and NMR spectroscopy (Figure S21). Variable temperature PXRD was also used to analyze **3D-CageHOF-1**, which showed that the material remained highly crystalline and stable up to 120 °C (Figures S22 and S23), in agreement with the differential scanning calorimetry (DSC) result that no visible phase changes were observed before 165 °C (Figure S24). We attribute the stability to a combination of the 3D hydrogen-bonded network and the additional π–π interactions between aromatic caps of the cages. In contrast to other HOFs, including triptycene-based HOFs with the same network topology,^16,26,27^ the bulky cage cores in **3D-CageHOF-1** play an essential role in obtaining a non-interpenetrated 3D structure.

To gain further information about the crystallization behavior of **Cage-3-NH**_**2**_, we carried out an *in situ* variable temperature PXRD experiment using the as-synthesized sample. We found that heating solvated crystals of **3D-CageHOF-1** from 298 to 363 K caused the structure to transform into the denser HOF-19 structure, with the PXRD patterns remaining the same after re-cooling the same to room temperature (Figure S25). However, activated crystals of **3D-CageHOF-1** were stable over the same temperature range (Figure S23), highlighting the importance of the crystallization solvent in facilitating the transformation of **3D-CageHOF-1** to HOF-19.

### Gas Sorption Analysis

**Figure 4:**
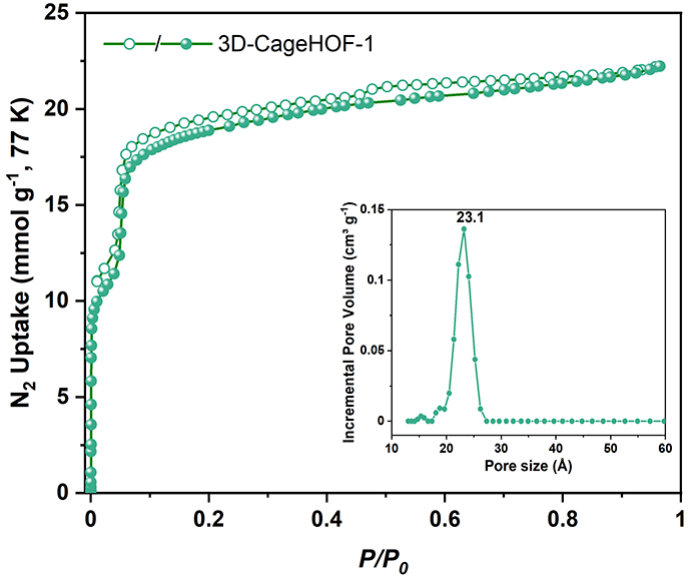
N_2_ sorption isotherms of **3D-CageHOF-1** at 77 K and pore size distribution (inset).

Encouraged by the apparent stability of the non-interpenetrated structure of **3D-CageHOF-1**, N_2_ sorption at 77 K was used to measure its porosity. The sorption isotherms of **3D-CageHOF-1** had a type-IV shape with a sharp uptake at low relative pressure (<0.01), followed by a step at *P*/*P*_0_ = 0.01–0.1 (Figures 4, S26–S29). The desorption hysteresis loop indicates that **3D-CageHOF-1** is mesoporous. This conclusion is supported by the derived pore size distribution plot that displays a narrow peak at 23 Å, close to the value of 22 Å based on the sc-XRD structure (Figure 4, inset).

Although it is challenging to find HOF building blocks that form structures with large pores without interpenetration, this shows that the use of organic cages as building blocks is one promising strategy. To our knowledge, **3D-CageHOF-1** is the first non-interpenetrating mesoporous 3D HOF with a stable skeleton (Table S6). **3D-CageHOF-1** exhibits a BET surface area of 1750 m^2^ g^–1^, which is about 2.5 times higher than HOF-19 (Figure S30).^33^ Furthermore, **3D-CageHOF-1** has a much lower structure density (0.54 g cm^–3^) than HOF-19 (1.00 g cm^–3^) (Figure S30).

## Conclusions

A cage-based HOF (**3D-CageHOF-1**) was discovered using computational CSP calculations and structural invariants to allow systematic comparisons between two CSP landscapes. In the mesoporous HOF phase, the bulky cage cores prevent network interpenetration, leading to the first example of a mesoporous non-interpenetrated 3D cage-HOF structure. **3D-CageHOF-1** has good structure stability after removing guest molecules from its pores, and its activated structure has a high BET surface area. The mesoporous structure **3D-CageHOF-1** could lead to new applications of HOFs in host–guest chemistry by enabling larger guests to occupy the crystal pores, such as enzymes.^56^

This study is also the first example of a molecular crystal that was computationally identified using a combination of CSP and structural invariants. Here, this led to the experimental discovery of a rare example of a mesoporous HOF. More broadly, we anticipate that this strategy will help to identify other functional molecular crystals by leveraging the proven power of structural analogy, supported by *a priori* lattice energy calculations and a formal metric for structural similarity between energy landscapes.

## Supporting Information Available

Computational methodology, structural invariants, synthetic procedures, PXRD, sc-XRD, DSC, TGA, and gas sorption analysis (PDF)

## Accession Codes

CCDC 2157172–2157173 contain the supplementary crystallographic data for this paper. These data can be obtained free of charge via www.ccdc.cam.ac.uk/data_request/cif, or by emailing data_request@ccdc.cam.ac.uk, or by contacting The Cambridge Crystallographic Data Centre, 12 Union Road, Cambridge CB2 1EZ, UK; fax: +44 1223 336033.
