# Supplementary material for: Analogy Powered by Prediction and Structural Invariants: Computationally Led Discovery of a Mesoporous Hydrogen-Bonded Organic Cage Crystal
Source: J Am Chem Soc. 2022 May 29;144(22):9893–901. doi: 10.1021/jacs.2c02653 (PMC9490843; doi:10.1021/jacs.2c02653)
Supplement: Supplementary file 1 — ja2c02653_si_001.pdf [file ja2c02653_si_001.pdf]

Supporting Information for

Analogy Powered by Prediction and Structural Invariants: Computationally-Led Discovery of a Mesoporous Hydrogen-Bonded Organic Cage Crystal

Qiang Zhu,[1,2] Jay Johal,[3] Daniel E. Widdowson,[4] Zhongfu Pang,[1,2] Boyu Li,[1] Christopher M. Kane,[1] Vitaliy Kurlin,[4*] Graeme M. Day,[3*] Marc A. Little,[1*] and Andrew I. Cooper[1,2*]

[1] Materials Innovation Factory and Department of Chemistry, University of Liverpool, 51 Oxford Street, Liverpool L7 3NY, UK.

[2] Leverhulme Research Centre for Functional Materials Design, University of Liverpool, 51 Oxford Street, Liverpool L7 3NY, UK.

[3] Computational Systems Chemistry, School of Chemistry, University of Southampton, SO17 1BJ, UK.

[4] Computer Science, University of Liverpool, Liverpool, L69 3BX, UK.

Contents

Section 1 Crystal Structure Prediction (CSP) 2

Section 2 New Isometry Invariants: Pointwise Distance Distributions (PDD) 12

Section 3 Experimental Material and Methods 19

Section 4 General Synthetic Procedures 21

Section 5 Crystallization of **Cage-3-NH$_2$** 24

Section 6 Gas Sorption Analysis of **3D-CageHOF-1** 39

Section 7 Single Crystal Data for **T2-ε** and **3D-CageHOF-1** 43

References 47

Section 1 Crystal Structure Prediction (CSP)

1.1 CSP Methods

The molecular structure of **Cage-3-NH$_2$** was extracted from the previously reported single-crystal structure of HOF-19,[1] and optimized by density functional theory (DFT) using Gaussian09.[2] The PBE0 functional[3] and 6-31G(d,p) basis sets were used along with GD3BJ empirical dispersion for the geometry optimization.[4] The final structure was checked by performing a frequency calculation at the same level of theory. CSP was performed using a quasi-random sampling method with the DFT-optimized molecular geometry held rigid throughout crystal structure generation and lattice energy minimization steps.[5] In the quasi-random search method, crystal structures are generated by mapping elements of a Sobol vector to the degrees of freedom defining a crystal structure (molecular positions, orientations and unit cell angles and lengths that are free to vary within the space group being considered). Initial crystal structures were checked for clashes between molecules. Clashes were detected between the convex hulls of each molecule. When these occurred, the unit cell was expanded to relieve clashes using the separating axis theorem. All accepted structures are lattice energy minimized: initially using a simple force field using atomic partial charges for the electrostatic model, then re-optimization using a force field with more detailed electrostatic interactions, using atomic multipoles.

The force field used for lattice energy minimization comprised the FIT empirically parametrized exp-6 repulsion-dispersion model [6] with electrostatics derived from the B3LYP/6-311G(d,p) charge density.[7] Atomic multipoles, up to hexadecapole on all atoms, were derived from a distributed multipole analysis, performed using GDMA.[8] Atomic partial charges were fitted to the molecular electrostatic potential derived from the atomic multipole model, using the MulFIT program.[9]

Crystal structures were generated with one molecule in the asymmetric unit (Z' = 1) within the 25 most common space groups: $Pbca$, $P2_1/c$, $P2_12_12_1$, $P\bar{1}$, $P2_1$, $C2/c$, $Pna2_1$, Cc, $Pca2_1$, $C2$, $P1$, $Pbcn$, Pc, $P2_12_12$, $P4_32_12$, $P4_1$, $P3_2$, $Fdd2$, $Pccn$, $P2/c$, $P6_1$, $I4_1/a$, $C222_1$, $P4_2/n$ and $R\bar{3}$. Trial crystal structures were generated, and energy minimized, until the target of 10,000 successfully

energy-minimized structures was reached in each space group. This took approximately 25 hours over 6 nodes (each with 40 cores) on the Iridis5 HPC facility at the University of Southampton. Duplicate crystal structures were removed from the final structure set by comparing predicted powder X-ray diffraction patterns (PXRD) of structures that are within 0.05 g cm^{-3} in density and within 1 kJ mol^{-1} in energy. PXRD patterns were compared using constrained dynamic time-warping. After duplicate removal, 11289 unique crystal structures were found.

Crystal structures for **T2** were taken from published work[10], which used the same force field as was applied for **Cage-3-NH$_2$**. Because the initial **T2** study[10] included a smaller set of space groups ($P2_1/c$, $P2_12_12_1$, $P\bar{1}$, $P2_1$, $C2/c$, $P4_2$), CSP was performed for **T2** within the space groups $Pbca$, $Pna2_1$, Cc, $Pca2_1$, $C2$, $P1$, $Pbcn$, Pc, $P2_12_12$, $P4_32_12$, $P4_1$, $P3_2$, $Fdd2$, $Pccn$, $P2/c$, $P6_1$, $I4_1/a$, $C222_1$, $P4_2/n$ and $R\bar{3}$ using the same procedure as for **Cage-3-NH$_2$**. Due to the inclusion of space group $P4_2$ in the **T2** dataset, a further CSP was performed for **Cage-3-NH$_2$** using the same method stated above for spacegroup $P4_2$. This gave a total of 11393 unique crystal structures for **Cage-3-NH$_2$** and 14497 for **T2** after duplicate removal from the sampling of 26 spacegroups.

Tight-binding DFT re-optimizations were performed on structures forming the leading edge of the energy-density distributions of crystal structures for **Cage-3-NH$_2$** and **T2**. The leading-edge structures were selected by dividing each distribution into a number of bins between its lowest and highest density structure and choosing a number of structures from each bin. By comparing the coverage of parameter choices for bins and structures for both distributions, 45 bins containing the 10 lowest energy structures in each bin were used to represent the leading edge of each distribution well.

The rigid CSP search prevents the molecules in the crystal structures from relaxing to more favorable geometries to maximize their interactions. The re-optimizations allow for this introduction of flexibility and the impact on the structures can be seen in Tables S1-2 and Figure S3.The implementation of third-order DFTB[11] in the DFTB+ software package[12] was used for the tight-binding DFT re-optimizations, using the LBFGS optimizer and the 3ob-3-1 Slater-Koster parameterization.[13] DFT-D3 Becke-Johnson damping was also used with parameters

taken from a published work.[14] The optimum k-point selection for the sampling of the Brillouin zone for each structure was determined and implemented using the Supercell Folding method into the calculations.

1.2 CSP Results

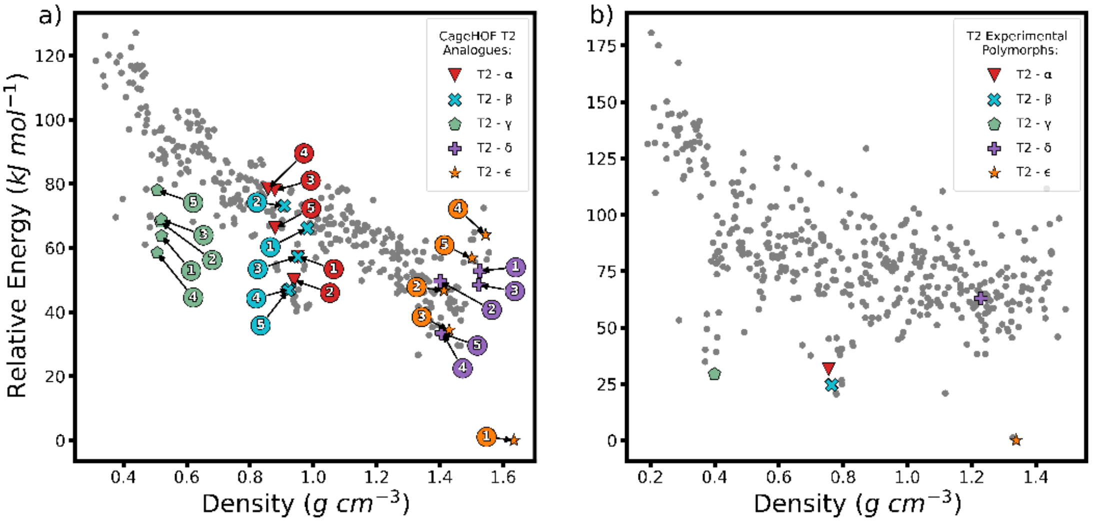

Figure S1. Energy-density distributions of the DFTB optimized leading edge structures of a) **Cage-3-NH$_2$**, and b) **T2**. The five closest pre-DFTB optimized analogues of each **T2** polymorph based on isometry invariants are highlighted on the **Cage-3-NH$_2$** landscape. The best matches to the known **T2** polymorphs are shown on the **T2** plot.

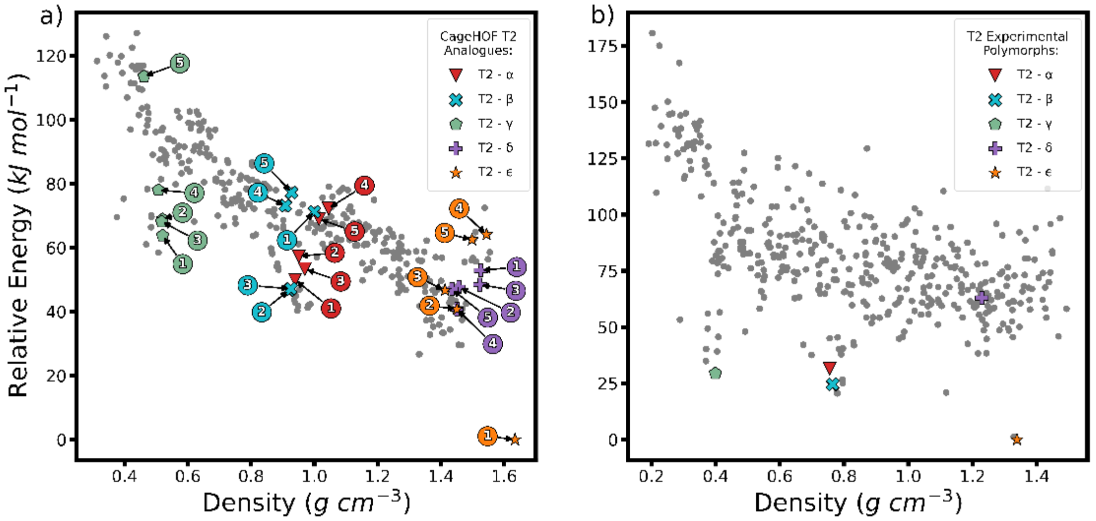

Figure S2. Energy-density distributions of the DFTB optimized leading edge structures of a) **Cage-3-NH$_2$**, and b) **T2**. The five closest DFTB optimized analogues of each **T2** polymorph based on isometry invariants are highlighted on the **Cage-3-NH$_2$** landscape. The best matches to the known **T2** polymorphs are shown on the **T2** plot.

Table S1. T2 and **Cage-3-NH₂** COMPACK comparisons for known polymorphs in the pre-DFTB optimized (rigid-molecule CSP) and DFTB optimized leading-edge structures. The searches were conducted by comparing molecular clusters of 30 molecules, with angle and distance tolerance of 20° and 20%, respectively, and ignoring hydrogen positions. All matches are 30/30 unless otherwise stated for HOF-19.

Structure	Pre-DFTB Optimised Structural Match	RMSD (Å)	DFTB Optimised Structural Match	RMSD (Å)
**T2-α**	ttbi-QR-86-23163-3	0.236	ttbi-QR-86-23163-3	0.225
**T2-β**	ttbi-QR-14-7540-3	0.829	ttbi-QR-14-7540-3	0.452
**T2-γ**	ttbi-QR-15-18932-3	0.243	ttbi-QR-15-18932-3	0.226
**T2-δ** [a]	ttbi-QR-2-5277-3	1.248	ttbi-QR-2-5277-3	1.239
**T2-ϵ**	ttbi-QR-2-5710-3	0.084	ttbi-QR-2-5710-3	0.069

**3D-CageHOF-1**	optcagehof-QR-19-7305-3	0.267	optcagehof-QR-19-7305-3	0.599
**HOF-19** [b]	optcagehof-QR-20-23336-3	0.825	optcagehof-QR-20-23336-3	0.298
	optcagehof-QR-14-15228-3	0.789	optcagehof-QR-14-15228-3	0.380

[a] Compack comparison of **T2** δ structure used increased angle and distance tolerances of 30° and 30 % respectively

[b] The HOF-19 CSP structural match to optcagehof-QR-20-23336-3 is the best match pre-DFTB. However, it is only a partial match with 29/30 molecules matching in the molecular clusters for both the pre-DFTB and post-DFTB structures. The optcagehof-QR-14-15228-3 structural match is poor pre-DFTB with 18/30 molecules matching in the molecular clusters, however post-DFTB it becomes a full 30/30 match.

Table S2. T2 and **Cage-3-NH_2** single molecule overlap RMSDs for the known experimental polymorphs to the gas phase optimized molecules for the pre-DFTB comparisons and to the structural matches shown in Table S1 post-DFTB calculated using COMPACK.

Structure	Pre-DFTB 1 Molecule RMSD (Å)	Post-DFTB Best Structural Match	Post-DFTB 1 Molecule RMSD (Å)
**T2**-α	0.093	ttbi-QR-86-23163-3	0.090
**T2**-β	0.379	ttbi-QR-14-7540-3	0.128
**T2**-γ	0.020	ttbi-QR-15-18932-3	0.032
**T2**-δ	0.429	ttbi-QR-2-5277-3	0.414
**T2**-ϵ	0.049	ttbi-QR-2-5710-3	0.028

**3D-CageHOF-1**	0.033	optcagehof-QR-19-7305-3	0.085
**HOF-19**	0.268	optcagehof-QR-14-15228-3	0.128
		optcagehof-QR-20-23336-3	0.172

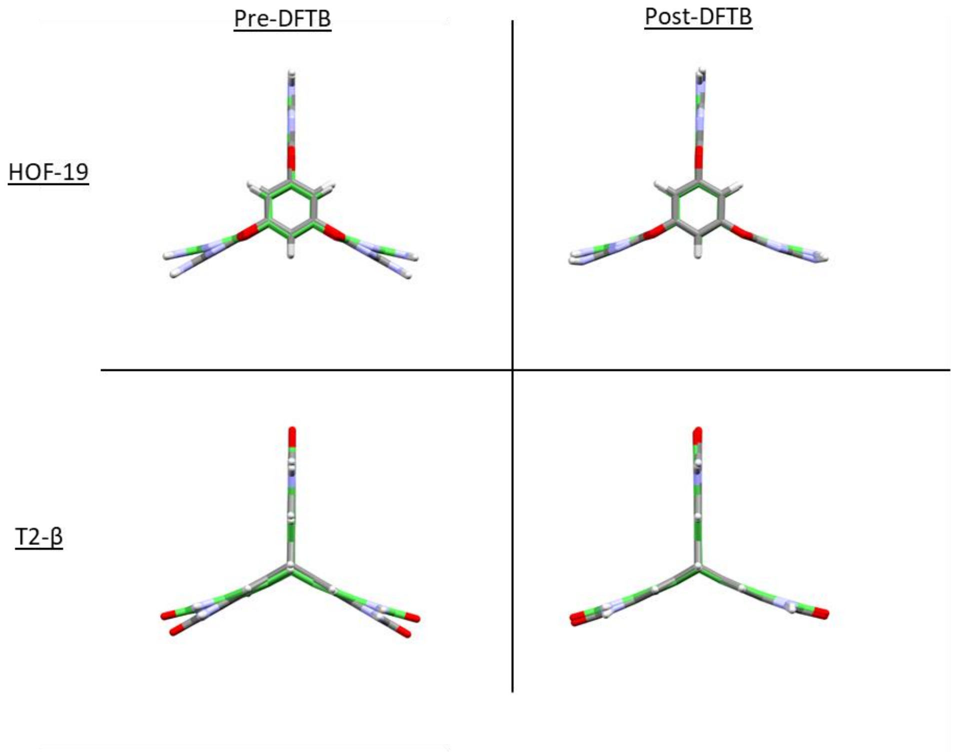

Figure S3. Single molecule overlaps for the experimental polymorphs HOF-19 and **T2**-β, both shown in green, to the gas phase optimized **Cage-3-NH$_2$** and **T2** molecules, respectively, pre-DFTB and their structural matches from Table S1 post-DFTB. The structural deviations from the gas phase optimized molecular geometries are apparent in these two crystal structures, and DFTB re-optimization improves the agreement of the molecular geometry with the experimental structures.

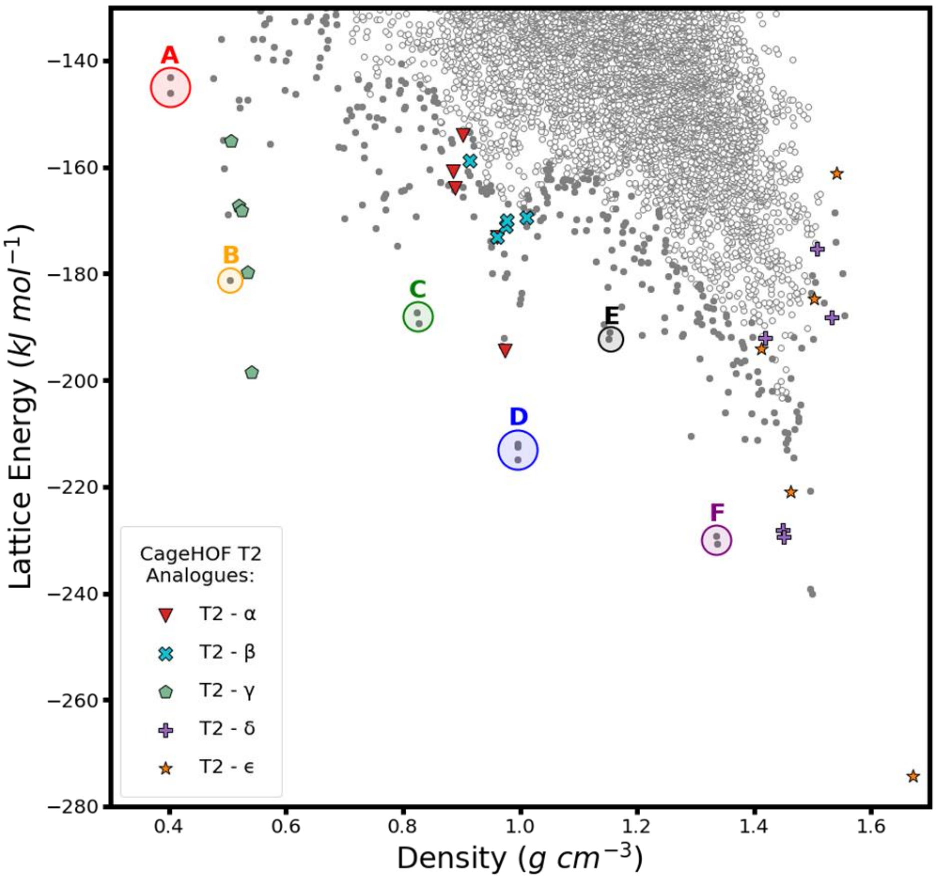

Figure S4. Energy-density distributions of the CSP structures for **Cage-3-NH_2** with the 5 closest analogues of each **T2** polymorph are highlighted in Figure 1. Other notable spikes on the landscape are circled and shown in Table S3 and Figure S5.

Table S3. Lowest energy structures located on the notable spikes from Figure S4 and their structural similarities to known polymorphs and CSP predicted crystal structures of **T2**.[15]

Spike Region	Structure	Density (g cm^{-3})	Lattice Energy (kJ mol^{-1})	T2 Structural Analogues [a]
A	optcagehof-QR-169-6056-3	0.4016	-146.13	Similarity to predicted Structure 1 of **T2** (see Figure S13 of ref 15)
A	optcagehof-QR-148-9622-3	0.4018	-143.15	Similarity to predicted Structure 1 of **T2** (see Figure S13 of ref 15)
B	optcagehof-QR-15-3198-3	0.5034	-181.17	Similarity to predicted structure **T2**-B (see Figure 3 of ref 15)
C	optcagehof-QR-14-3136-3	0.8273	-189.23	Similarity to **T2**-β
C	optcagehof-QR-29-3150-3	0.8235	-187.32	Similarity to **T2**-β
D	optcagehof-QR-15-23755-3	0.9963	-214.80	Similarity to **T2**-β
D	optcagehof-QR-15-11739-3	0.9964	-212.51	Similarity to **T2**-β
D	optcagehof-QR-20-31475-3	0.9952	-211.91	Similarity to **T2**-β
E	optcagehof-QR-148-1612-3	1.1518	-192.19	Similarity to **T2**-A (see Figure 3 of ref 15)
E	optcagehof-QR-148-6152-3	1.1524	-190.94	Similarity to **T2**-A (see Figure 3 of ref 15)
F	optcagehof-QR-148-11488-3	1.3378	-230.78	Similarity to **T2**-A (see Figure 3 of ref 15)
F	optcagehof-QR-148-1358-3	1.3349	-229.22	Similarity to **T2**-A (see Figure 3 of ref 15)

[a] **T2** Structure 1, **T2**-A and **T2**-B all refer to CSP predicted crystal structures from reference 15.

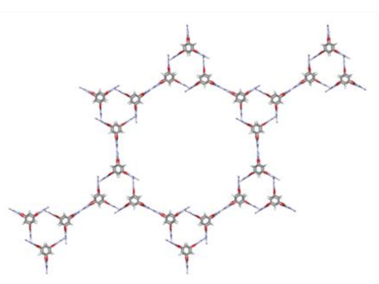

A - optcagehof-QR-169-6056-3

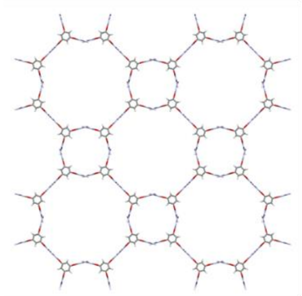

B - optcagehof-QR-15-3198-3

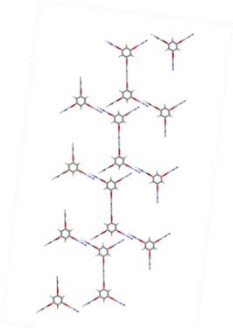

C - optcagehof-QR-14-3136-3

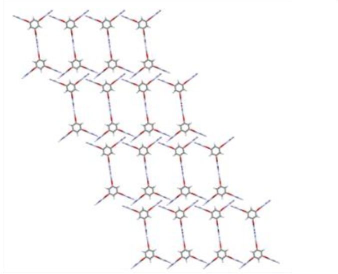

D - optcagehof-QR-15-23755-3

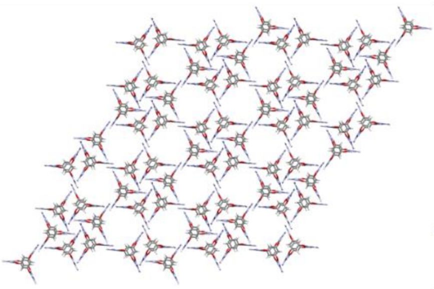

E - optcagehof-QR-148-1612-3

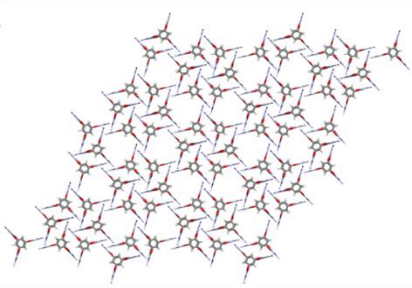

F - optcagehof-QR-148-11488-3

Figure S5. Lowest energy structure for each spike region from Table S3.

Section 2 New Isometry Invariants: Pointwise Distance Distributions (PDD)

To compare structures on the **T2** and **Cage-3-NH$_2$** landscapes, we used new isometry invariants, which continuously quantify the similarity of any two crystals using geometry.

An *isometry* is any composition of translations, rotations or reflections. An isometry *invariant* of a crystal does not change under isometries applied to the input. Therefore, it is independent of transformations that do not affect the rigid structure of the crystal, as well as superfluous changes in a representation like extensions of the unit cell.[16]

The new isometry invariants are defined for any *periodic sets*, given by a finite motif of points that repeats periodically according to a lattice. A crystal structure can give rise to a periodic set either by taking a point in the center of each atom or the center of mass of each molecule. For this cross-landscape comparison where structures have significantly different molecules, the latter is more appropriate as the resulting invariant mostly ignores differences arising from the different molecules.

To construct the *Pointwise Distance Distribution* (PDD) invariant of a periodic set S with points $p_1, ..., p_m$ in a unit cell, we first find for each motif point p_i the row of ordered distances $d_{i1} \leq d_{i2} \leq \ldots \leq d_{ik}$ to the first k nearest neighbors of p_i in the infinite periodic set S.

Two ordered rows of distances can be *lexicographically* compared as in a dictionary: a row $(d_{i1}, d_{i2}, ..., d_{ik})$ is less than another row $(d'_{i1}, d'_{i2}, ..., d'_{ik})$ if, comparing two rows coordinate-wise, we find a strictly smaller distance in the former row, so $d_{ij} < d'_{ij}$ for some index j from 1 to k.

After lexicographically sorting the rows, if any group of w rows are identical, they are replaced with one row, and the weight w/m is applied (so unique rows have weight $1/m$). Finally, the weights are canonically placed in the extra first column, providing a matrix with $k + 1$ columns and no more than m rows.

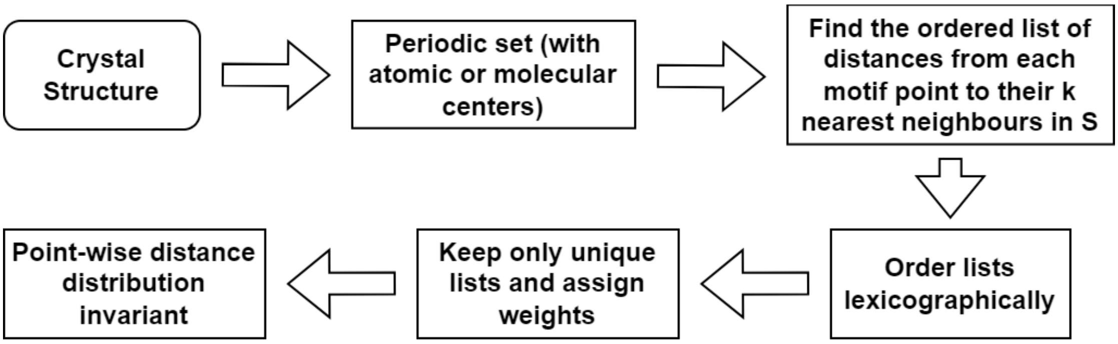

Figure S6. Workflow of calculating the Pointwise Distance Distribution (PDD) of a crystal.

The resulting matrix is an isometry invariant,[17] which is also a complete at least of general positions, meaning that almost any two non-isometric periodic sets have different PDDs for large enough *k*.[17] We have no counter-examples to completeness and conjecture that PDD is complete for all periodic sets of points.

To define a proper distance between PDDs, a base distance between rows of two PDDs (without weights) is required. This could be any metric between vectors. In the experiments, we used L_∞, the maximum absolute difference between any corresponding elements of rows. Once a distance between rows is chosen, the Earth Mover's Distance[18] compares the weighted distributions of rows, finding an optimal matching between rows while respecting the weights, and the 'cost' for this optimal matching is a proper EMD metric satisfying all metric axioms and also the continuity under perturbations of points.[17] This distance has units of Angstroms, being a weighted sum of differences of inter-point distances.

An invariant of periodic sets is continuous if small perturbations to the input points always result in a small distance between outputs. This continuity is needed to capture the notion of similarity between non-isometric structures, but is not satisfied by other invariants such as the reduced cell and is impossible with discrete invariants such as space groups, as in Figure S7 below.

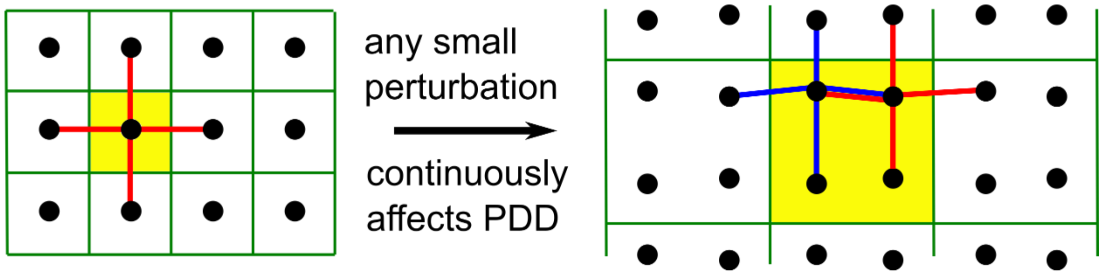

$$\begin{pmatrix} 1 \,|\, 1 & 1 & 1 & 1 \end{pmatrix} \longrightarrow \begin{matrix} \text{EMD} = \\ 0.102 \end{matrix} \longleftarrow \left(\begin{array}{c|cccc} 1/2 & 0.8 & 1.005 & 1.005 & 1.2 \\ 1/2 & 1 & 1 & 1.005 & 1.005 \end{array}\right)$$

Figure S7. A unit square lattice has its unit cell extended and one point slightly moved, resulting in different PDDs with a small Earth Mover's distance, illustrating continuity under perturbations.

Figure S7 shows a unit square lattice with a point at (0.5,0.5) whose PDD (k = 4) consists of one row with the first four equal distances 1, 1, 1, 1, and a perturbed set obtained by extending the unit cell and moving one point, giving the motif (0.5,0.4), (0.5,1.6), (1.5,0.5), (1.5,1.5) in a square 2 x 2 cell. For the perturbed set, the initial matrix of distances has two pairs of identical rows: (0.8,1.005,1.005, 1.2) in blue and (1,1,1.005,1.005) in red. The final PDD has the two above rows, each with a weight of 0.5. In this case, the Earth Mover's distance (with k = 4) is equal to the average L_∞ distance between the initial row and two perturbed rows, so EMD = 0.5 (|0.8-1| + |1.005-1|) = 0.102.

$$\begin{pmatrix} 0.8 & 1.005 & 1.005 & 1.2 \\ 0.8 & 1.005 & 1.005 & 1.2 \\ 1 & 1 & 1.005 & 1.005 \\ 1 & 1 & 1.005 & 1.005 \end{pmatrix} \longrightarrow \left(\begin{array}{c|cccc} 1/2 & 0.8 & 1.005 & 1.005 & 1.2 \\ 1/2 & 1 & 1 & 1.005 & 1.005 \end{array}\right)$$

Figure S8. The four motif points of the perturbed set in Figure S7 give two repeated sets of distances because of the reflectional symmetry in the horizontal line cutting the unit cell in half. Since both rows appear twice out of four total, they both have the weight 2/4 = 1/2.

Continuity was important in this context, as experimentally determined structures were compared with simulated ones and because a slight difference in molecular diameter led to differences in the extended packing of analogous structures across the landscapes.

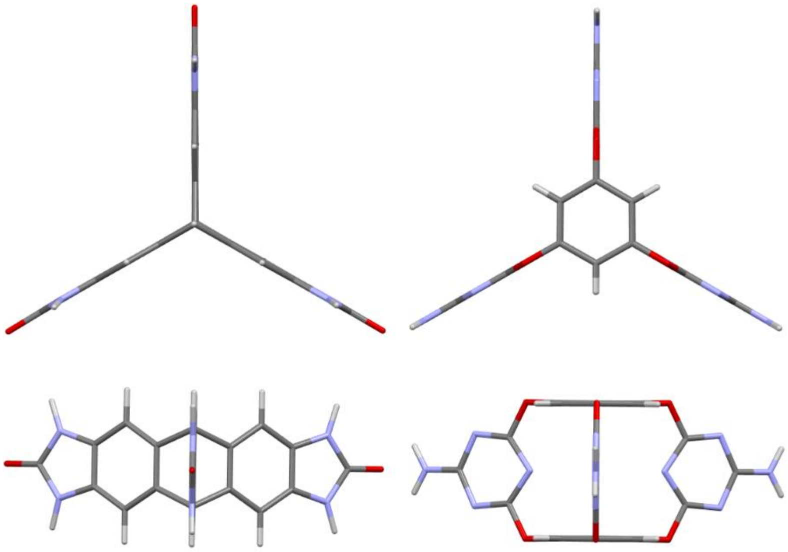

Figure S9. Side-by-side view of **T2** (left) and **Cage-3-NH$_2$** (right) molecules. Crystals were represented with a point at centre of mass of each molecule to capture the similarity between structures whose molecules are different but pack in similar ways.

The PDD of a crystal is defined for any positive integer k, and adding more columns does not affect previous values. The asymptotic behavior of PDD as k becomes infinitely large is well understood and depends only on the crystal's (point) density;[16] the distinguishing behavior for a crystal occurs for early values of k. It is, therefore, appropriate to choose a reasonable, non-specific value of k, to capture enough intermolecular interactions.

To find cross-landscape analogues, each structure was represented as a periodic set with a point in the center of mass (COM) of each molecule. We calculated the Earth Mover's Distance between PDDs (with k = 100) of the experimental **T2** structures and structures in the leading edge of the **Cage-3-NH$_2$** landscape. Pairs with the smallest distances were manually inspected to determine if an analogue had been found. We found that four out of five **T2** polymorphs had one or more analogous structures in the simulated **Cage-3-NH$_2$** landscape, shown in Figure S10 below.

Finding crystal structures with similar COM arrangements but different molecular orientations cannot be ruled out theoretically. However, we did not find any examples of such pairs in or between the CSP datasets in this study.

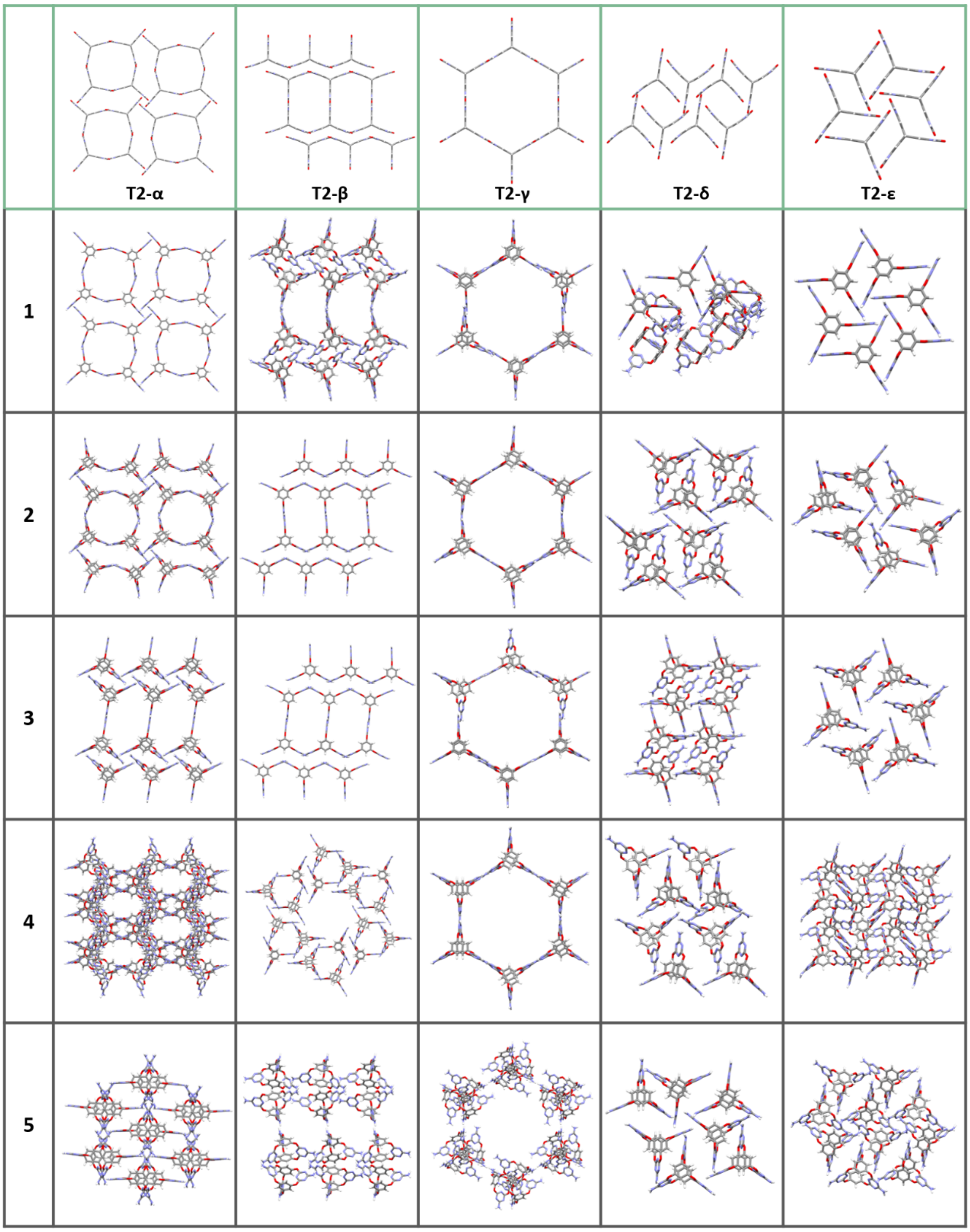

Figure S10. Closest matches to experimental **T2** crystals in the DFTB-optimized leading edge of the **Cage-3-NH$_2$** CSP landscape, measured with Earth Mover's distance between PDDs (k = 100) on molecular centers. Some inexact matches were expected due to the simplification of taking molecular centers, and the difference in diameter between the **T2** and **Cage-3-NH$_2$** molecules.

Table S4. Table of distances (row in blue) and relative structure ID from experimental **T2** polymorphs to their 10 nearest neighbors in the pre-DFTB optimized leading edge of the **Cage-3-NH$_2$** CSP landscape, measured with Earth Mover's distance between PDDs (k = 100) on molecular centers.

	**T2-α**	**T2-β**	**T2-γ**	**T2-δ**	**T2-ε**
**1**	optcagehof-QR-88-10964-3	optcagehof-QR-14-13326-3	optcagehof-QR-148-1342-3	optcagehof-QR-148-13812-3	optcagehof-QR-148-11368-3
	1.142197	1.326365	1.743697	1.1938	0.90891
**2**	optcagehof-QR-86-21274-3	optcagehof-QR-148-2802-3	optcagehof-QR-43-474-3	optcagehof-QR-14-3657-3	optcagehof-QR-2-10657-3
	1.214197	1.424895	1.93119	1.25004	2.417236
**3**	optcagehof-QR-14-3022-3	optcagehof-QR-88-10964-3	optcagehof-QR-56-22795-3	optcagehof-QR-15-25453-3	optcagehof-QR-14-7411-3
	1.6265	1.451267	2.011587	1.361269	2.491841
**4**	optcagehof-QR-60-9337-3	optcagehof-QR-43-24182-3	optcagehof-QR-19-7305-3	optcagehof-QR-18-15793-3	optcagehof-QR-14-8584-3
	1.633095	1.458763	2.203599	1.3624	2.50604
**5**	optcagehof-QR-13-15047-3	optcagehof-QR-14-2319-3	optcagehof-QR-169-12312-3	optcagehof-QR-18-4695-3	optcagehof-QR-148-11154-3
	1.672457	1.470991	2.338594661	1.36404	2.566225
**6**	optcagehof-QR-60-24103-3	optcagehof-QR-14-4505-3	optcagehof-QR-60-24199-3	optcagehof-QR-15-5617-3	optcagehof-QR-14-6779-3
	1.687658	1.471067	2.544114	1.367849	2.576922
**7**	optcagehof-QR-15-12941-3	optcagehof-QR-61-19364-3	optcagehof-QR-148-1754-3	optcagehof-QR-43-825-3	optcagehof-QR-14-12323-3
	1.698217	1.484675	2.665126	1.371332	2.586541
**8**	optcagehof-QR-15-11739-3	optcagehof-QR-61-12954-3	optcagehof-QR-13-23069-3	optcagehof-QR-148-18963-3	optcagehof-QR-148-5262-3
	1.702426	1.486792	2.787179	1.43363	2.588573
**9**	optcagehof-QR-15-4406-3	optcagehof-QR-15-23749-3	optcagehof-QR-43-8500-3	optcagehof-QR-14-14882-3	optcagehof-QR-14-14882-3
	1.717827	1.5006	2.840228	1.474998	2.604298
**10**	optcagehof-QR-14-3850-3	optcagehof-QR-169-5242-3	optcagehof-QR-56-22029-3	optcagehof-QR-15-18377-3	optcagehof-QR-43-825-3
	1.721662	1.502415498	2.873307	1.496392	2.615008

Table S5. Table of distances (row in blue) and relative structure ID from experimental **T2** polymorphs to their 10 nearest neighbors in the DFTB optimized leading edge of the **Cage-3-NH$_2$** CSP landscape, measured with Earth Mover's distance between PDDs (k = 100) on molecular centers.

	**T2-α**	**T2-β**	**T2-γ**	**T2-δ**	**T2-ε**
**1**	optcagehof-QR-86-21274-3	optcagehof-QR-56-17462-3	optcagehof-QR-148-1342-3	optcagehof-QR-148-13812-3	optcagehof-QR-148-11368-3
	0.916238	1.387153	1.769997	1.192342	1.330857669
**2**	optcagehof-QR-88-10964-3	optcagehof-QR-14-2319-3	optcagehof-QR-56-22795-3	optcagehof-QR-15-5617-3	optcagehof-QR-43-825-3
	1.041285	1.398905	1.94708	1.420931	2.3582355
**3**	optcagehof-QR-14-4505-3	optcagehof-QR-43-24182-3	optcagehof-QR-43-474-3	optcagehof-QR-15-25453-3	optcagehof-QR-2-10657-3
	1.538665	1.408535	2.035902	1.424535	2.484133
**4**	optcagehof-QR-15-12941-3	optcagehof-QR-148-2802-3	optcagehof-QR-169-12312-3	optcagehof-QR-43-825-3	optcagehof-QR-14-8584-3
	1.688699	1.416784	2.348987	1.435755	2.495804
**5**	optcagehof-QR-60-24103-3	optcagehof-QR-61-19364-3	optcagehof-QR-148-1754-3	optcagehof-QR-148-18963-3	optcagehof-QR-14-6779-3
	1.708619	1.449245	2.647033	1.438976	2.5539815
**6**	optcagehof-QR-43-24182-3	optcagehof-QR-14-4505-3	optcagehof-QR-56-17928-3	optcagehof-QR-15-5249-3	optcagehof-QR-148-11154-3
	1.769934	1.470869	2.669579	1.464557	2.576743423
**7**	optcagehof-QR-169-5242-3	optcagehof-QR-169-5242-3	optcagehof-QR-60-24199-3	optcagehof-QR-15-23836-3	optcagehof-QR-14-12323-3
	1.795626	1.485339	2.695075	1.465528	2.583503
**8**	optcagehof-QR-60-9337-3	optcagehof-QR-2-11556-3	optcagehof-QR-60-24730-3	optcagehof-QR-18-4695-3	optcagehof-QR-148-5262-3
	1.834637	1.502784	2.785893	1.476703	2.618336715
**9**	optcagehof-QR-15-19386-3	optcagehof-QR-15-21559-3	optcagehof-QR-43-8500-3	optcagehof-QR-14-14882-3	optcagehof-QR-14-7411-3
	1.836055	1.510513	2.812405	1.477998	2.634451
**10**	optcagehof-QR-15-11739-3	optcagehof-QR-88-10964-3	optcagehof-QR-56-22029-3	optcagehof-QR-14-3657-3	optcagehof-QR-14-13819-3
	1.836489	1.510623	2.885253	1.4833	2.666112

Section 3 Experimental Material and Methods

All reagents were obtained from Sigma-Aldrich, TCI Europe, Fisher, and Alfa Aesar and used as received. Anhydrous solvents were purchased from Acros Organics and used without further purification. All gases for sorption analysis were supplied by BOC at a purity of ≥99.9%. Reactions were carried out under a nitrogen atmosphere using standard Schlenk techniques.

3.1 Single Crystal X-ray Diffraction (SC-XRD)

T2 was synthesized using a previously reported procedure.[15] The single crystal of **T2**-ε was grown by sublimation at *ca.* 800-850 °C using a tube furnace under reduced pressure (~3.5 x 10^{-2} mbar). The single crystal data was measured on Rigaku MicroMax-007 HF rotating anode diffractometer (Mo-Kα radiation, λ = 0.71073 Å, Kappa 4-circle goniometer, Rigaku Saturn 724+ detector). Empirical absorption corrections, using the multi-scan method, were performed with the program SADABS.[22,23] The structure was solved by SIR2004,[24] and refined by full-matrix least-squares on $|F|^2$ by SHELXL,[20] interfaced through the programme OLEX2.[21] All non-H atoms were refined anisotropically, and C-H H-atoms were fixed in geometrically estimated positions and refined using the riding model. For full refinement details, see Table S7, and for images of the crystal structure, see Figure S31.

Single crystal X-ray data for **Cage-3-NH_2** was measured on a Rigaku MicroMax-007 HF rotating anode diffractometer (Mo-Kα radiation, λ = 0.71073 Å, Kappa 4-circle goniometer, HyPix-6000HE detector) and data reduction was performed using the CrysAlisPro software. Structures were solved with SHELXT[19] and refined by full-matrix least-squares on $|F|^2$ by SHELXL,[20] interfaced through the programme OLEX2.[21] All non-H atoms were refined anisotropically, and all H-atoms were fixed in geometrically estimated positions and refined using the riding model. For full refinement details, see Table S8, and for images of the crystal structure, see Figure S32.

3.2 PXRD

Laboratory powder X-ray diffraction PXRD data patterns were collected in transmission mode on samples held on thin Mylar film in aluminium well plates on a Panalytical Empyrean diffractometer equipped with a high throughput screening (HTS) XYZ stage, X-ray focusing

mirror, and PIXcel detector, using Cu-Kα radiation. Capillary PXRD patterns were collected on powdered samples loaded in borosilicate glass capillaries, and the capillaries were spun to improve averaging. For the variable temperature experiments, the capillaries were then heated from room temperature to 175 °C using an Oxford Cryosystems 700 series cryostream. An equilibration time of 1 hour was used at each temperature set point.

3.3 NMR

NMR spectra were recorded on a Bruker 400 NMR spectrometer at 400 MHz (^{1}H) and 100 MHz (^{13}C) and referenced against the residual ^{1}H or ^{13}C signal of the solvent.

3.4 Gas Sorption Analysis

Surface areas were measured by nitrogen sorption at 77.3 K. Powder samples were degassed offline, followed by degassing on the analysis port under vacuum at 25 °C for 15 hours. Isotherm measurements were performed using a Micromeritics 3flex surface characterization analyzer, equipped with a Cold-Edge technologies liquid helium cryostat chiller unit for temperature control.

3.5 Thermogravimetric Analysis (TGA)

TGA analysis was carried out using a Netzsch TG 209 F1 Libra instrument using a Al_2O_3 crucible. The sample was heated at a rate of 20 °C/min from 25 °C to 900 °C under a dry nitrogen gas flow.

3.6 Differential Scanning Calorimetry (DSC) Analysis

DSC measurements were carried out on a Discovery DSC instrument with an auto sampler. The sample was heated in a sealed Tzero Aluminum Hermetic pan at a rate of 20 °C/min from 25 °C to 250 °C.

3.7 Optical Microscopy Images

Optical microscopy images were recorded using an Olympus BX53 Microscope with 10X objective lenses and Olympus DP26 digital color camera under a reflection model.

Section 4 General Synthetic Procedures

Synthesis of 1,3,5-tris((4,6-dichloro-1,3,5-triazin-2-yl)oxy)benzene (HCTAB)

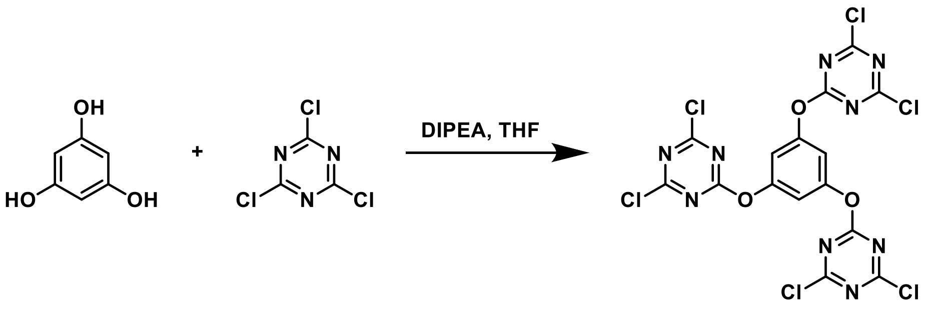

1,3,5-tris((4,6-dichloro-1,3,5-triazin-2-yl)oxy)benzene (HCTAB) was synthesized based on the previous methods with slight modification.[25]

To a solution of cyanuric chloride (16.59 g, 90 mmol) in THF (150 mL), a mixture of phloroglucinol (2.52 g, 20 mmol) and *N*, *N*-diisopropylethylamine (DIPEA, 13.6 mL, 78 mmol) in THF (100 mL) was added dropwise over a period of 2 h at 0 °C. The reaction was stirred for another 4 h at 0 °C and overnight at room temperature. After filtration and concentration, the crude product was collected and further purified by chromatography using CH_2Cl_2/ petroleum ether = 30-80% as eluent to afford the product as a white solid in a yield of 54%: 6.1 g (10.7 mmol).

^{1}H NMR (400 MHz, $CDCl_3$): δ 7.17 (s, 6H); ^{13}C NMR (100 MHz, $CDCl_3$): δ 173.5, 170.4, 151.7, 113.7.

Synthesis of Cage-3-Cl

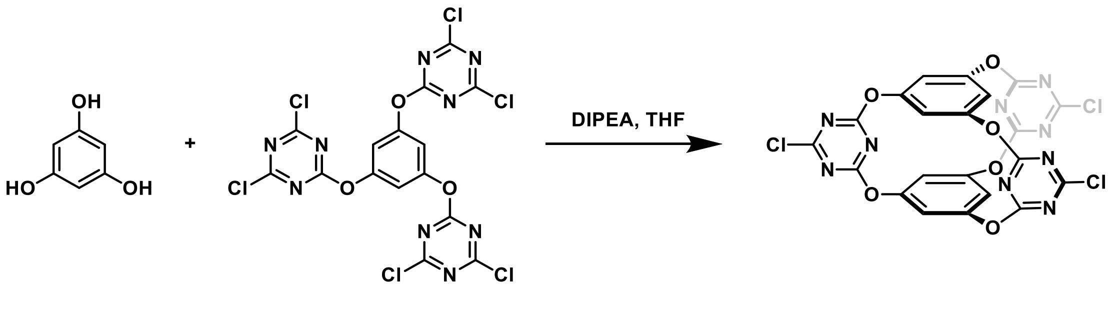

To a mixture of DIPEA (2.36 mL, 14.4 mmol) in acetone (400 mL) at 0 °C, a solution of phloroglucinol (2.28 g, 4 mmol) in acetone (200 mL), and a solution of HCTAB (2.28g, 4 mmol) in acetone (200 mL), were added dropwise at the same rate over 4 h. After the addition was complete, the reaction was stirred at room temperature for 96 h. The solvent was then removed by rotary evaporation, and the crude product was purified by chromatography using

acetone/CH_2Cl_2 (2% acetone by volume) as eluent to afford the product as a white solid in a yield of 33%: 620 mg (1 mmol).

^{1}H NMR (400 MHz, $CDCl_3$): δ 6.69 (s, 6H); ^{13}C NMR (100 MHz, $CDCl_3$): δ 175.0, 172.6, 153.0, 115.0.

Synthesis of Cage-3-NH₂

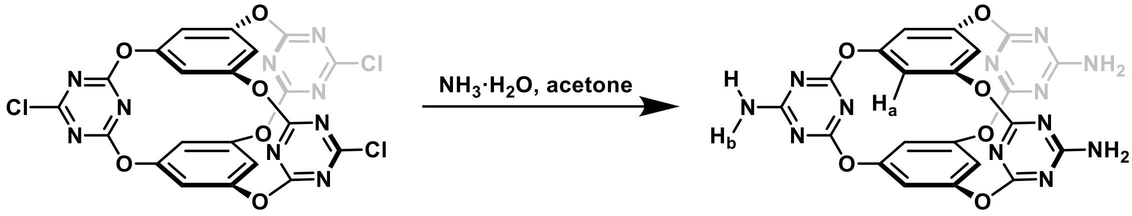

Cage-3-NH_2 was synthesized based on literature with a slight modification.[11]

To a solution of Cage-3-Cl (870 mg, 1.48 mmol) in acetone (45 mL), ammonium hydroxide (0.36 mL, 35%) was added. The resulting mixture was stirred for 20 h at room temperature. The white solid was obtained by filtration and washed with acetone. Then the crude product was dispersed in the solution of ammonium hydroxide (0.2 mL) in acetone/H_2O (20 mL / 4 mL) and stirred for another 12 h to afford the product as a white solid by filtration in a yield of 64%: 500 mg (0.95 mmol).

^{1}H NMR (400 MHz, DMSO-d_6): δ 7.76 (s, 6H_a), 6.71 (s, 6H_b); ^{13}C NMR (100 MHz, DMSO-d_6): δ 172.5, 170.5, 153.2, 115.0.

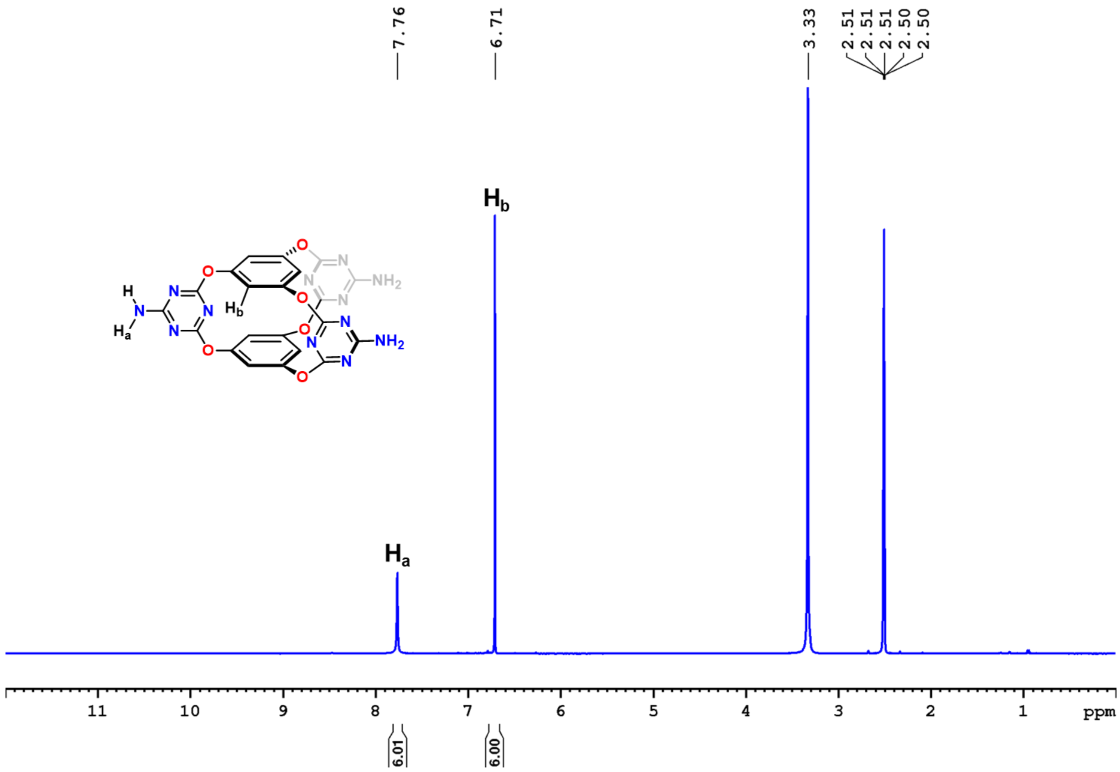

Figure S11. ^{1}H NMR (400 MHz, DMSO-d_6) spectrum of **Cage-3-NH$_2$**.

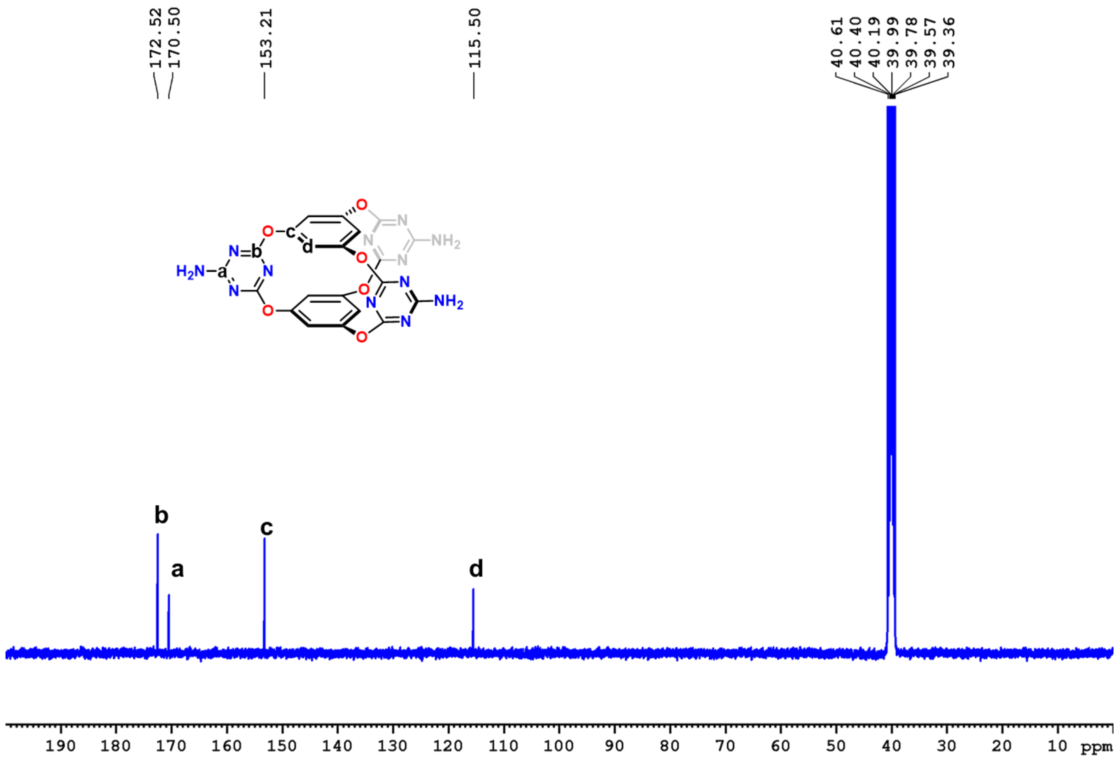

Figure S12. ^{13}C NMR (100 MHz, DMSO-d_6) spectrum of **Cage-3-NH$_2$**.

Section 5 Crystallization of Cage-3-NH_2

10 mg **Cage-3-NH_2** was dissolved in the mixture of formic acid (10 mL) and aniline (10 uL, 1 molar eq per **Cage-3-NH_2**), and sonicated for 5-10 min. The solution was filtered into two 15 mL glass vials (15 mL) through a 0.45 um PTFE syringe filter. The vials were then placed in a sealed chamber with diethyl ether and left undisturbed overnight at room temperature, which afforded needle-like single crystals suitable for single crystal X-ray diffraction analysis. Before gas sorption analysis, the crystallization solvents were exchanged with acetone ten times, and the crystals were then activated with supercritical CO_2.

HOF-19 can be obtained using the method reported in the literature.[11]

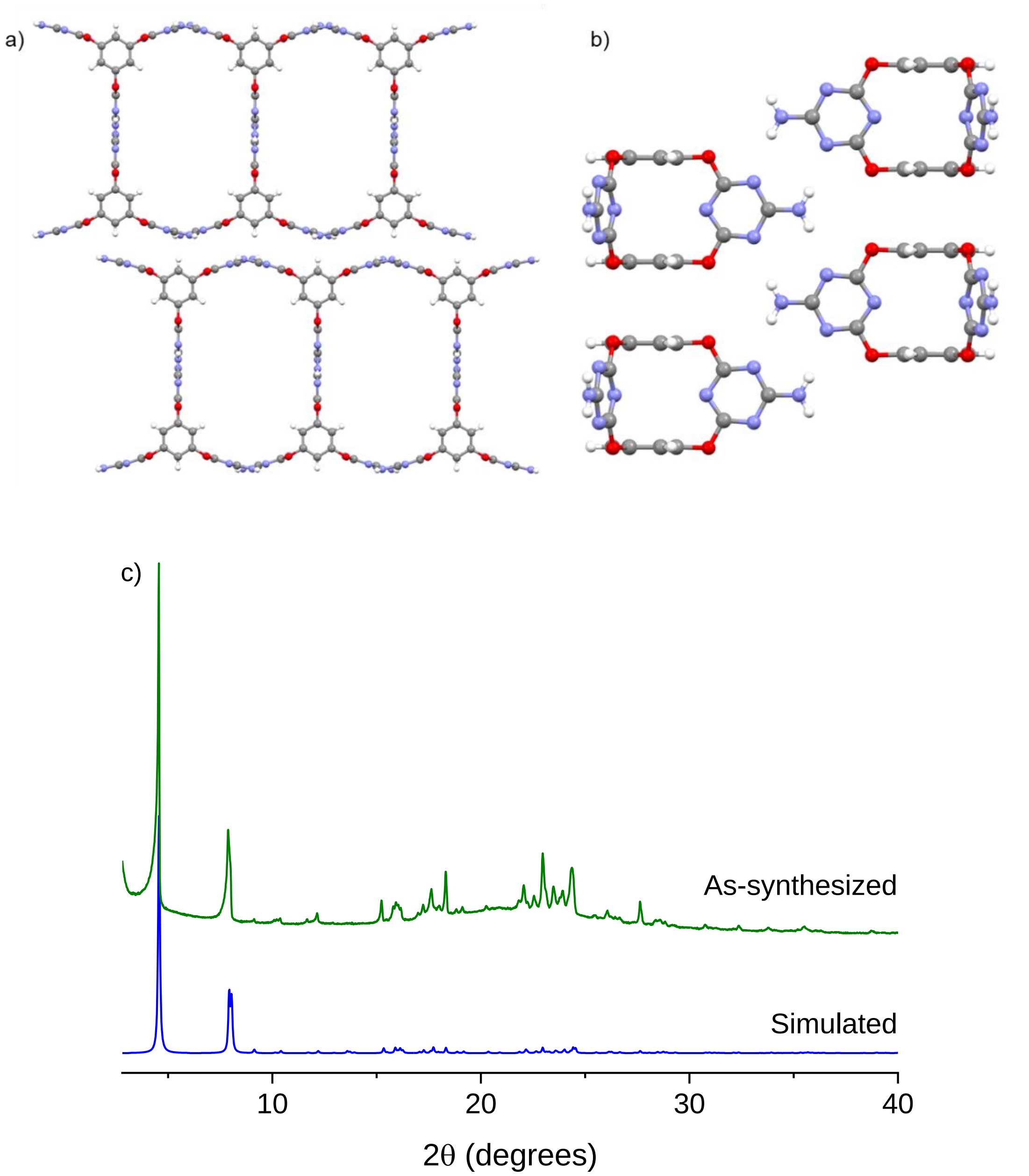

Figure S13. Crystal structures of HOF-19 [11]. Front view (a) and top view (b) of **Cage-3-NH$_2$** in the sc-XRD structure of HOF-19, Single-crystal atom colours: C, grey; N, blue; O, red; H, white. (c) Comparison between the experimental PXRD pattern of as-synthesized HOF-19 that was grown from the mixture of HCOOH and **Cage-3-NH$_2$** by slow evaporation and the simulated PXRD pattern based on the sc-XRD structure.

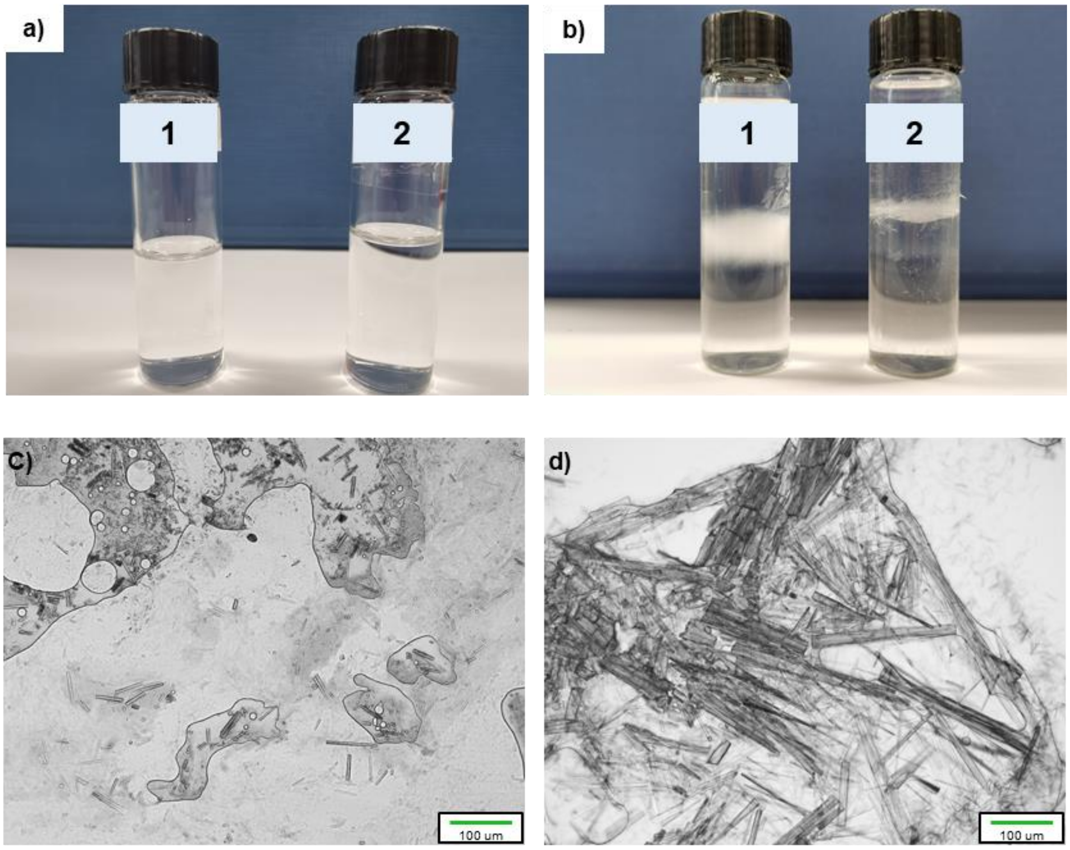

Figure S14. Images of **3D-CageHOF-1** crystals showing optimization of the crystallization conditions. a) Photographs of crystallizations containing no aniline in formic acid (**1**) and 1 molar eq of aniline per **Cage-3-NH$_2$** in formic acid (**2**) at the beginning of the crystallization. b) Photographs of crystallizations containing no aniline (**1**) and 1 molar eq of aniline per **Cage-3-NH$_2$** (**2**) after 24 h at room temperature. After 24 h, we found that vial **1** contained a thick layer of thin, needle-shaped crystals. In contrast, a thinner layer of needle-shaped crystals that appeared thicker by eye was observed in vial **2**. Despite the difference in crystal size, crystals from vials (**1**) and (**2**) samples had the same crystal structure by PXRD (see Figure S15). Optical microscope images of, c) needle-shaped crystals found in vial **1** after 36 h, and d) needle-shaped crystals found in vial **2** after 36 h. A 100 um scale bar is included in the inserts. The thicker needle-shaped crystals in vial **2** indicate that aniline slowed down the crystallization processes. A crystal suitable for sc-XRD analysis was grown using the same approach by diffusing diethyl ether vapour into a mixture of **Cage-3-NH$_2$** in formic acid (1 mg mL^{-1}) containing 1 molar eq aniline per **Cage-3-NH$_2$** as the modulator.

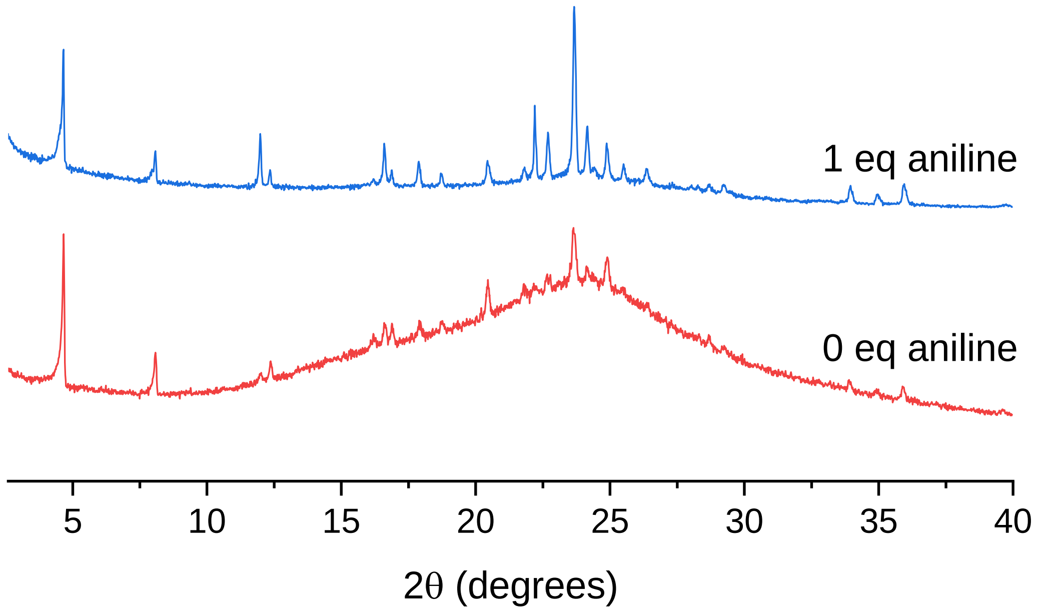

Figure S15. PXRD patterns for solvated crystals grown from **Cage-3-NH$_2$** in formic acid with 0 eq and 1 molar eq of aniline per **Cage-3-NH$_2$** in formic acid. The crystal grown without aniline were smaller (see Figure S14) and more challenging to load, and, as a result, a more significant solvent background was observed.

Table S6. Summary of structure properties of porous HOFs with 3D frameworks.

	Void (%)	Pore size (Å)	SA_{BET} ($m^2\ g^{-1}$)	Pore type	Ref.
HOF-1a	42	8.2[a]	359	microporous	26
SOF-1a	34	7.8[a]	474	microporous	27
TTBI	60	7.8, 10[b]	2796	microporous	28
d-POS-1	31.5	10.9[b]	398	microporous	29
NPSU-3	16.1	10.8[c]	29	microporous	30
HOF-2	54.3	4.8[a]	238	microporous	31
HOF-4	42.5	3.8 x 4.1[a]	312	microporous	32
SOF-7a	48	13.6[b]	900	microporous	33
HOF-3	75	7[a]	165	microporous	34
HOF-5a	41.1	3.9 x 5.5, 3.9 x 6.8[a]	1101	microporous	35
HOF-6	63.4	6.4, 7.5[a]	130	microporous	36
Cyclotetrabenzoin	10	-	42	microporous	37
Tcpb	38	18.5[b]	1095	microporous	38
IISERP-HOF1	34	9.1 x 9.4[c]	412	microporous	39
Benzo-trisimidazole	21.7	2.9 x 4.6[c]	131	microporous	40
CPHAT-1a	31	6.7[c]	649	microporous	41
HOF-TCBP	56	17.8 x 26.3[c]	2066	microporous	42
**T2**-γ	-	19.9[c]	3425	microporous	15
HOF-9a	22	~3.4 x 8.8[c]	286	microporous	43
FDM-15	-	12.5[b]	749	microporous	44
HOF-11	33.2	6.2 x 6.8[c]	687	microporous	45
TCF-1	17.3	2.9 x 5.5, 4.2 x 6.4[c]	-	microporous	46
CBPHAT-1a	45	12[b]	1288	microporous	47
CPOS-1	-	-	216	microporous	48
CPOS-2	-	-	129	microporous	
CPOS-3	-	-	12	microporous	

CPOS-4	-	-	29	microporous	
PETHOF-1	80	11[b]	1150	microporous	49
PETHOF-2	49	11[b]	1140	microporous	
PETHOF-3	80	13[b]	600	microporous	50
CPOS-5	14.6	5.3 x 6.8[c]	-	microporous	51
JLU-SOF-2	43.7	5.6, 3.8[c]	937	microporous	52
JLU-SOF-3	51.3	5.8, 3.2[c]	1141	microporous	
HOF-76a	-	8.5[b]	1121	microporous	53
ZJU-HOF-1	-	5-9[b]	1465	microporous	54
PFC-11	35.2	-	751	microporous	55
PFC-12	35.2	-	654	microporous	
PFC-13	34.6	-	-	microporous	
HOF-FJU-1.	20	6.0, 11.4[b]	385	microporous	56
UPC-H4a	-	-	210	microporous	57
UPC-HOF-6	-	5.7 x 9.4, 2.8 x 3.6[c]	237	microporous	58
HOF1	35.4	6.5[b]	465	microporous	59
1-Ni	70	-	1239	microporous	60
1-Co	70	-	1192	microporous	
PFC-2[d]	68.1	10.2, 29.2[b]	1014	meso-microporous	61
HOF-16	22.5	6.7[c]	279	microporous	62
HOF-20a	42.1	12.7[b]	1323	microporous	63
HOF-30a	20	4.2[c]	361	microporous	64
**3D-CageHOF-1**	72.5	23[b]	1750	mesoporous	**This work**

[a] Distance of atom centers including vdW radii;

[b] Pore size distribution calculated based on the gas sorption isotherms;

[c] Calculated by using a molecule probe (1.2 Å);

[d] PFC-2 has a 5-fold interpenetrated **nbo** topology.

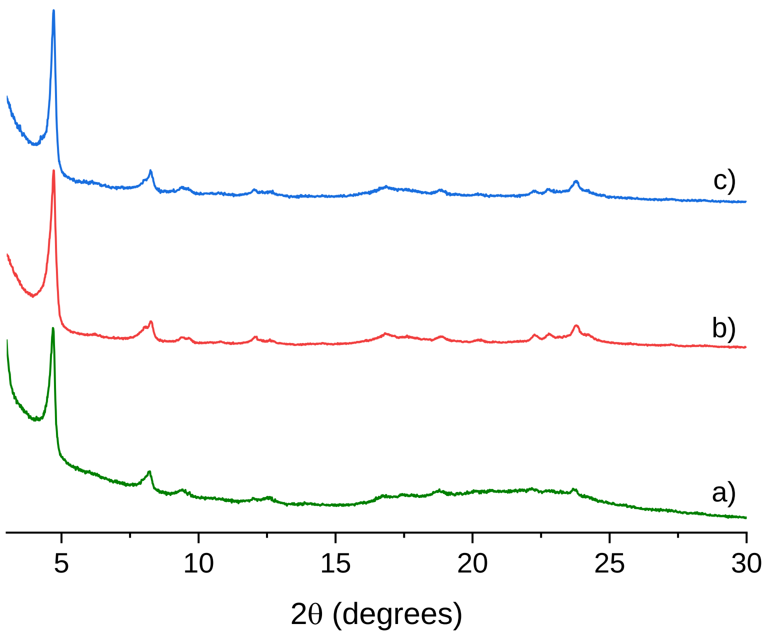

Figure S16. PXRD patterns of desolvated **3D-CageHOF-1** grown using different conditions. a) as grown from a mixture of **Cage-3-NH$_2$** (15 mg), HCOOH (15 mL) and aniline (1 molar eq per **Cage-3-NH$_2$**) by the vapour diffusion of diethyl ether; b) grown from a mixture of **Cage-3-NH$_2$** (15 mg), HCOOH (6 mL) and aniline (1 molar eq per **Cage-3-NH$_2$**) by the vapour diffusion of diethyl ether; c) grown from a mixture of **Cage-3-NH$_2$** (15 mg), HCOOH (6 mL) and aniline (1 molar eq per **Cage-3-NH$_2$**) by the vapour diffusion of acetone. The crystallization solvents in all the as-synthesized **3D-CageHOF-1** samples were exchanged with acetone after replacing the acetone solvent ten times, and the samples were then dried under N_2.

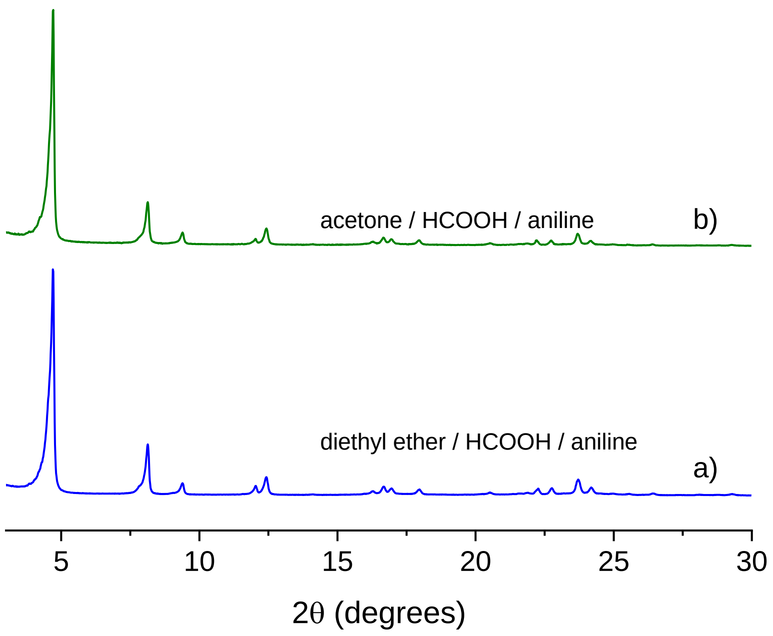

Figure S17. PXRD patterns of desolvated **3D-CageHOF-1** grown using different conditions. a) grown from a mixture of **Cage-3-NH₂** (15 mg), HCOOH (15 mL) and aniline (1 molar eq per **Cage-3-NH₂**) by the vapour diffusion of diethyl ether; b) grown from a mixture of **Cage-3-NH₂** (15 mg), HCOOH (6 mL) and aniline (1 molar eq per **Cage-3-NH₂**) by the vapor diffusion of acetone. All the as-synthesized samples were exchanged with acetone ten times and then activated under sc-CO_2.

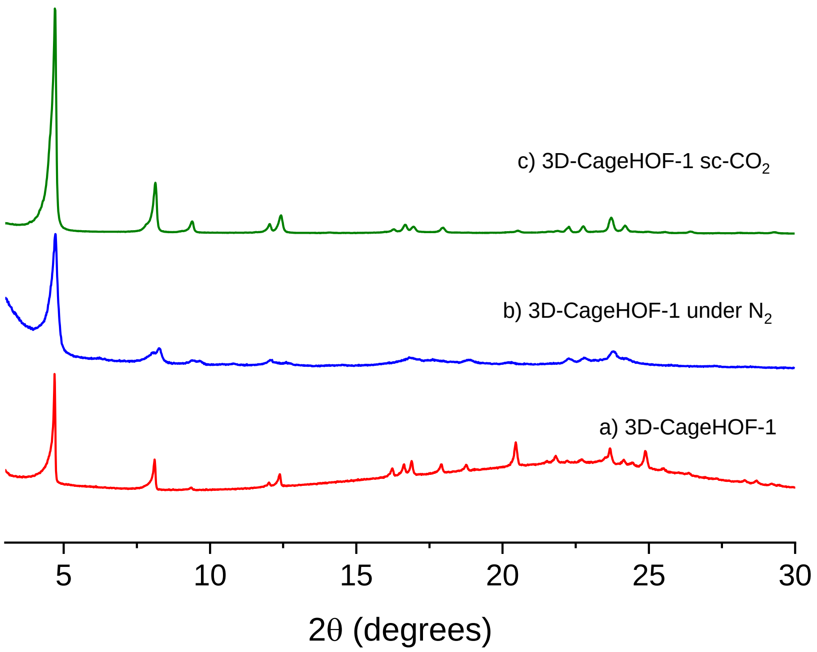

Figure S18. PXRD patterns of **3D-CageHOF-1** that were recorded after attempting different activation methods. The crystallization solvents in the as-synthesized **3D-CageHOF-1** were exchanged with acetone after replacing the acetone solvent ten times, and the samples were then dried under N_2 (b) or by sc-CO_2 (c).

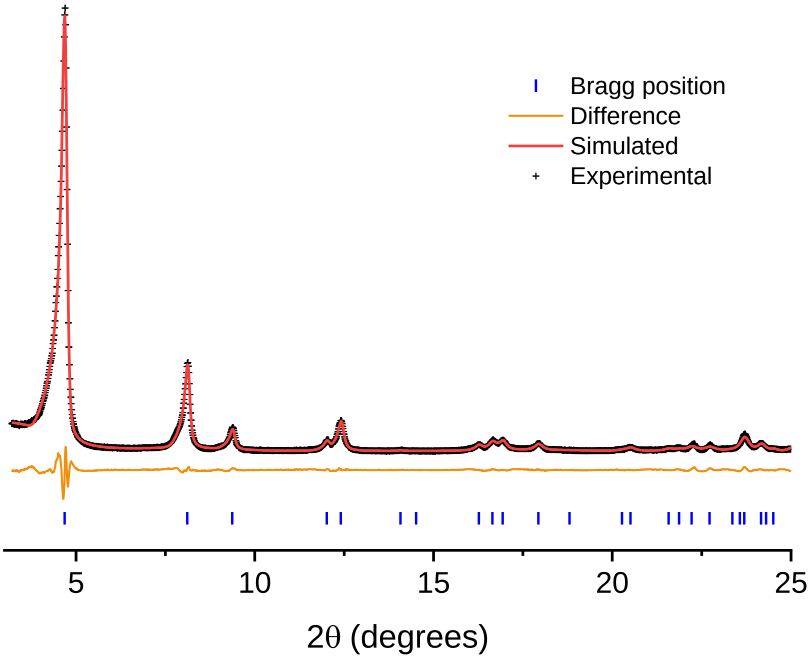

Figure S19. Pawley fit for the PXRD pattern of activated **3D-CageHOF-1**. Black cross: experimental PXRD pattern, red line: fitting pattern, yellow curve: difference between experimental and refinement, blue bars: reflection positions, Cu-Kα, R_{wp} = 6.6%, R_p = 5.1%, χ^2 = 2.15 ($P6_3/mmc$, $a = b$ = 21.779 Å, c = 7.995 Å, V = 3284 Å^3).

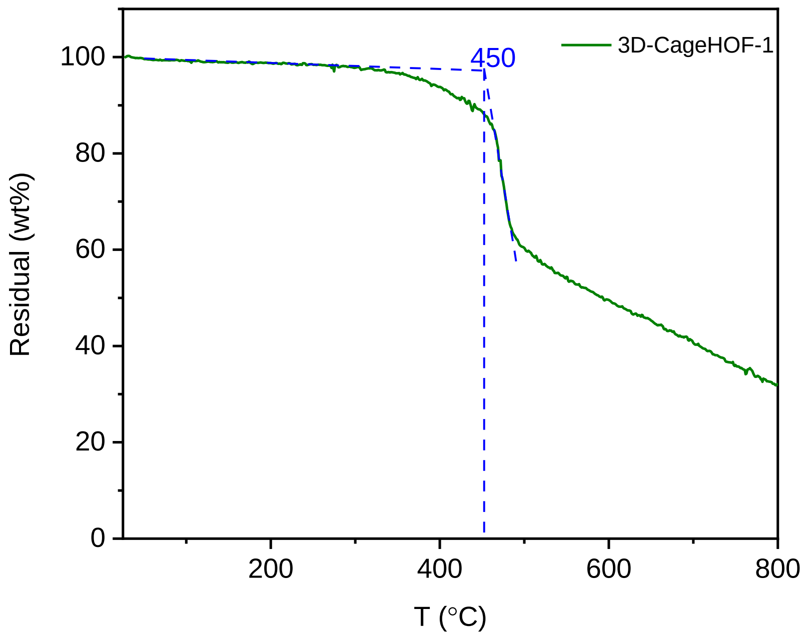

Figure S20. TGA plot of **3D-CageHOF-1** recorded after desolvation of the crystal pores.

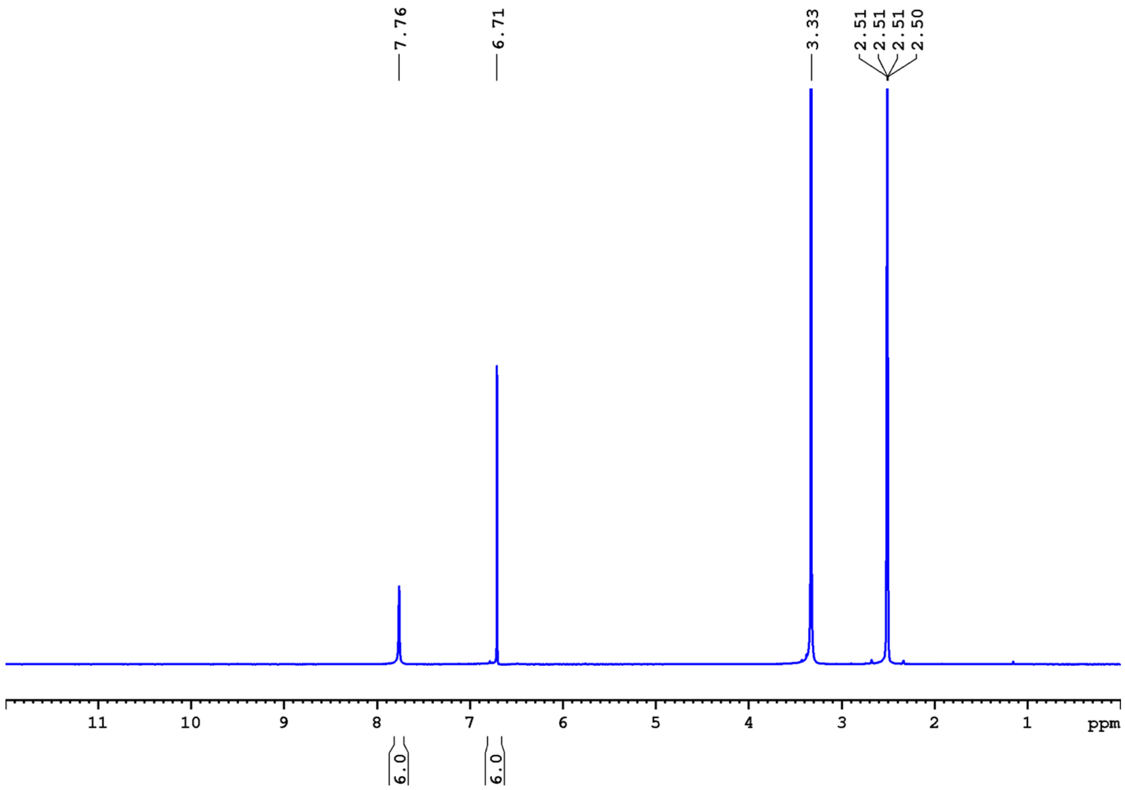

Figure S21. ^{1}H NMR (400 MHz, DMSO-d_6) spectrum of **Cage-3-NH$_2$** recorded after desolvation of the crystal pores. The NMR sample was prepared by completely dissolving the sample of **3D-CageHOF-1** in DMSO-d_6.

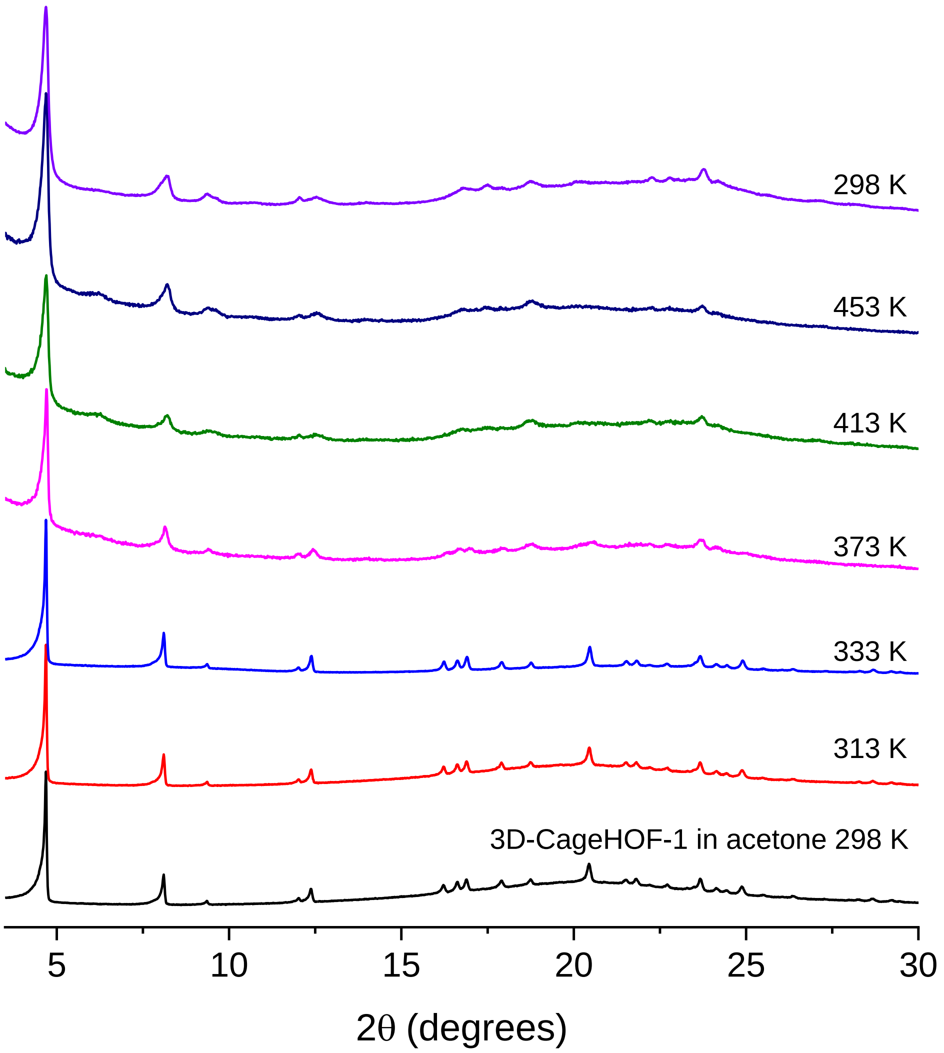

Figure S22. A stacked plot of VT-PXRD patterns that were recorded during the heating of an acetone solvated sample of **3D-CageHOF-1**. The **3D-CageHOF-1** sample was grown from the mixture of aniline (1 molar eq per **Cage-3-**NH_2) and **Cage-3-**NH_2 in formic acid by the vapour diffusion of diethyl ether. The capillary was left to equilibrate for 1 h at each temperature before each PXRD pattern was recorded.

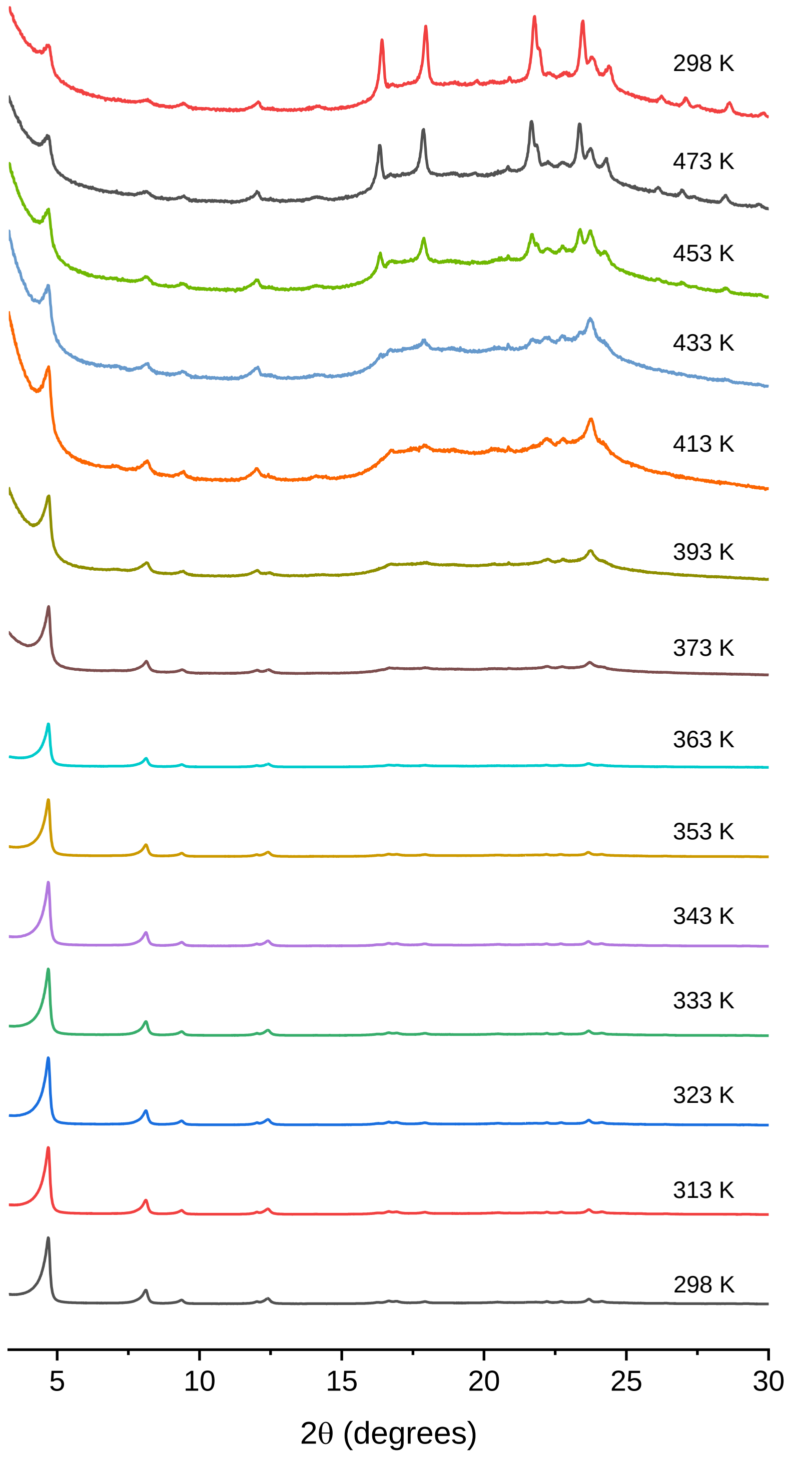

Figure S23. A stacked plot of VT-PXRD patterns that were recorded after heating an sc-CO_2 activated sample of **3D-CageHOF-1**. The **3D-CageHOF-1** sample was grown from the mixture of aniline (1 molar eq per **Cage-3-NH$_2$**) and **Cage-3-NH$_2$** in formic acid by the vapour diffusion of diethyl ether. The capillary was left to equilibrate for 1 h at each temperature before each PXRD pattern was recorded.

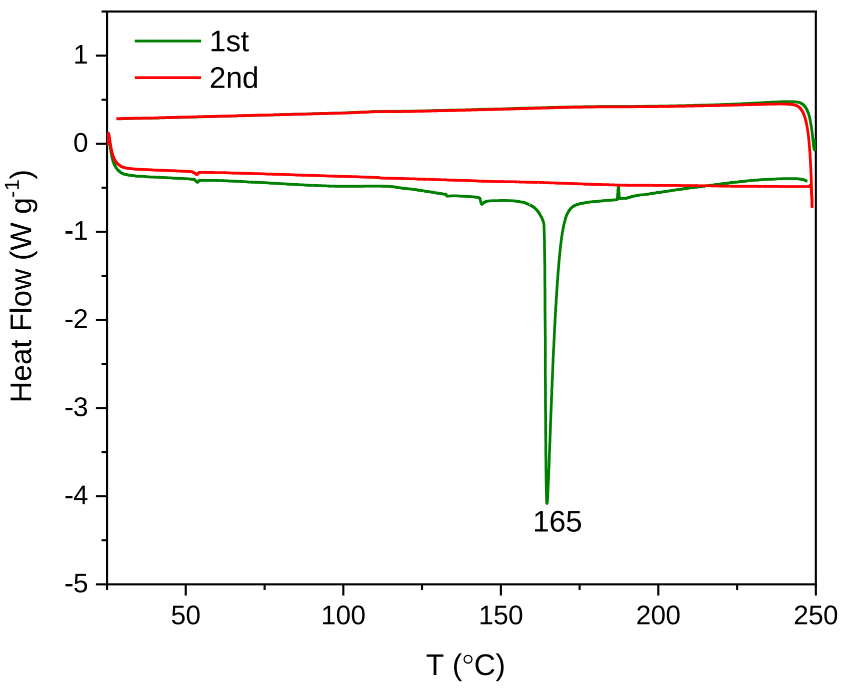

Figure S24. DSC plot for **3D-CageHOF-1**. The sample was analysized after $scCO_2$ activation. There was no evidence of a phase transformation in the DSC plot until 165 °C, which is consistent with the VT-PXRD results.

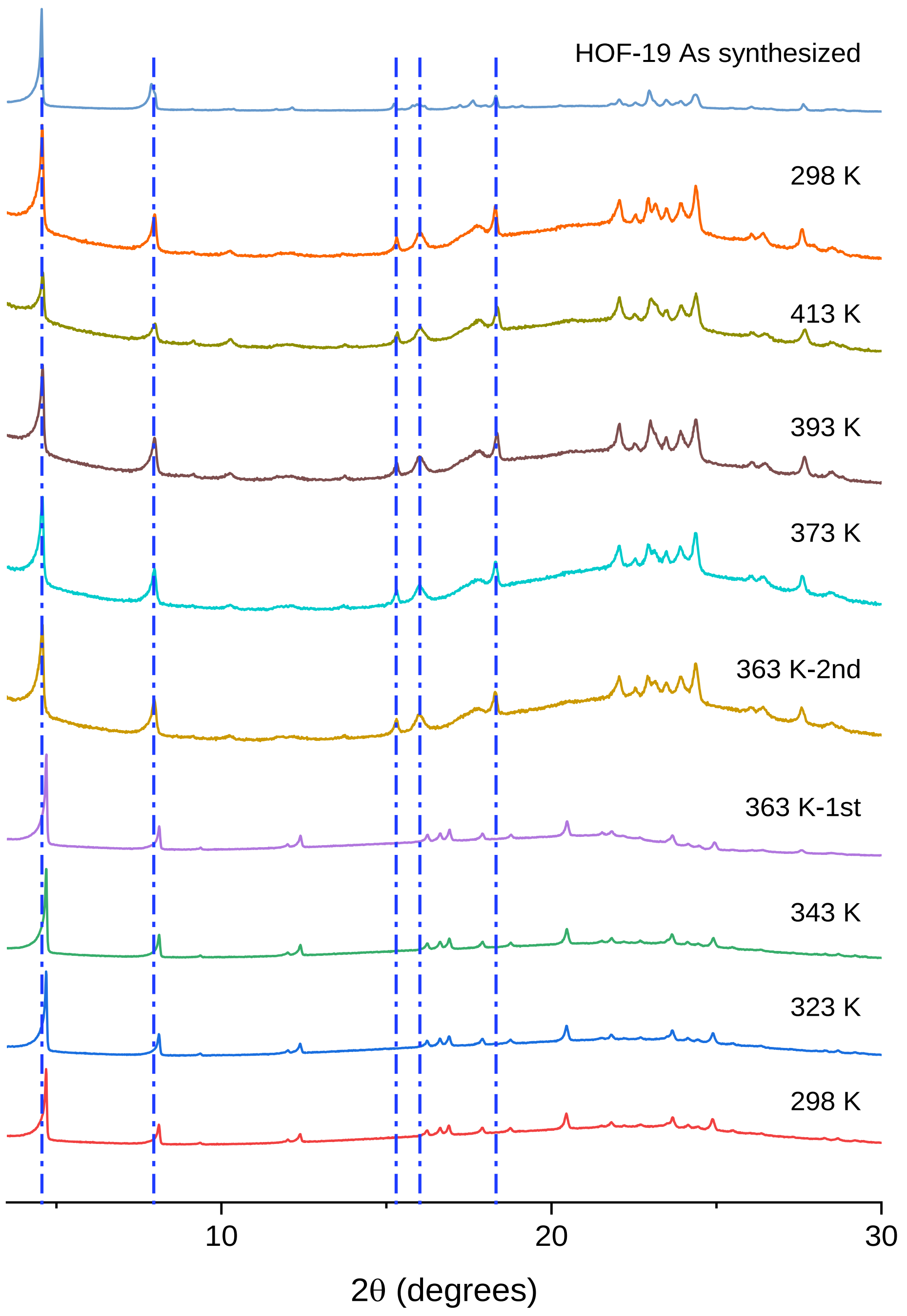

Figure S25. A stacked plot of variable temperature PXRD (VT-PXRD) patterns that were recorded during the heating and then cooling of an as-synthesized sample of **3D-CageHOF-1**. The PXRD patterns are stacked from top to bottom. The **3D-CageHOF-1** sample was grown from the mixture of aniline (1 molar eq per **Cage-3-NH$_2$**) and **Cage-3-NH$_2$** in formic acid by the vapour diffusion of diethyl ether. The capillary was left to equilibrate for 1 h at each temperature before each PXRD pattern was recorded.

Section 6 Gas Sorption Analysis of 3D-CageHOF-1

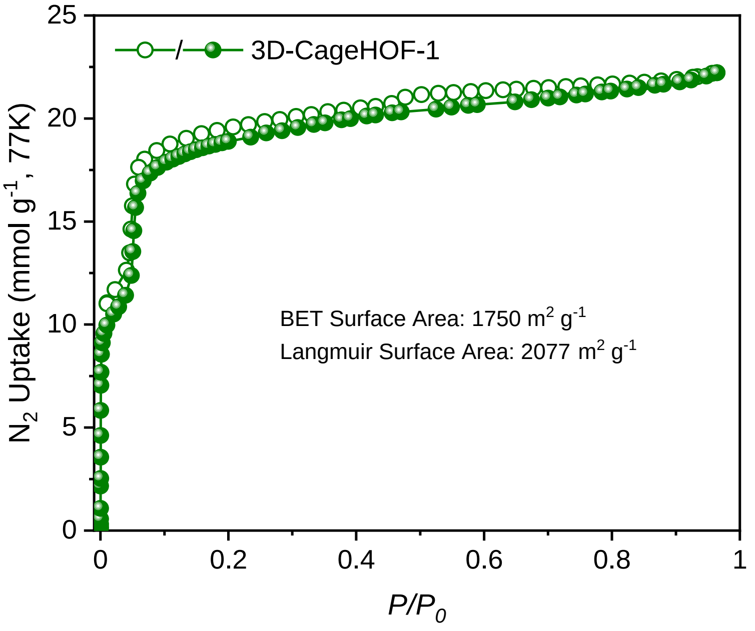

Figure S26. N_2 sorption isotherm of **3D-CageHOF-1** at 77 K. The sample was activated with $scCO_2$ before this analysis. Closed symbols: adsorption points, open symbols: desorption points.

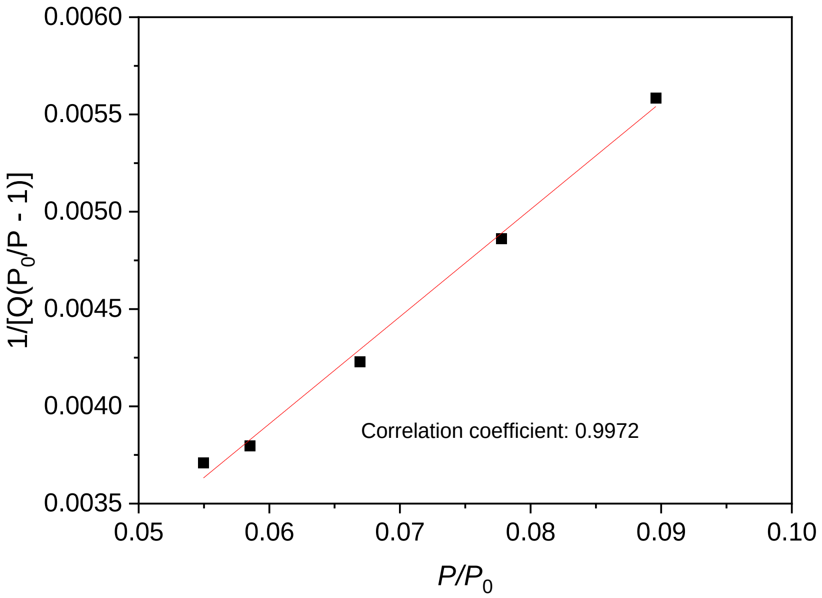

Figure S27. t-Plot for BET surface area calculation.

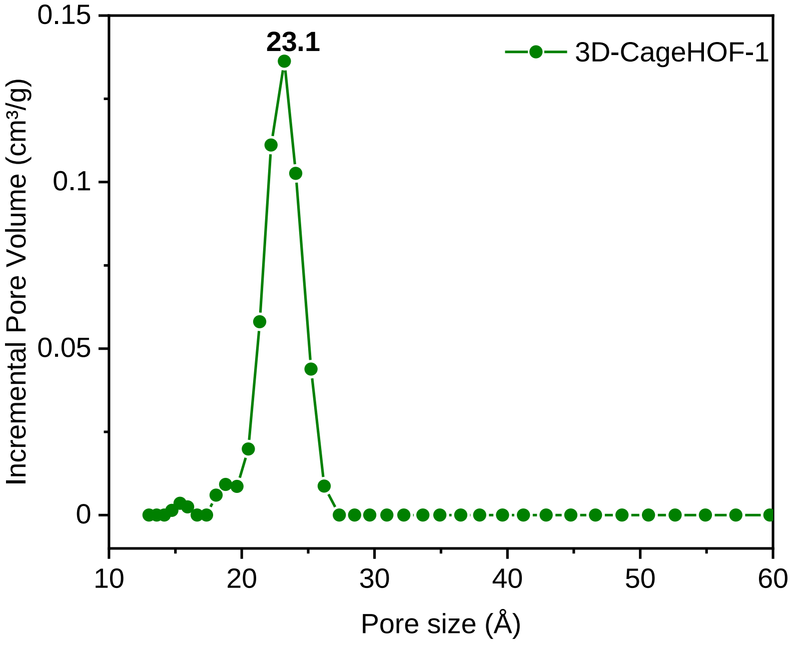

Figure S28. Pore size distribution plot for **3D-CageHOF-1** that was calculated using the N_2@77-Carbon Cylindrical Pores, SWNT model.

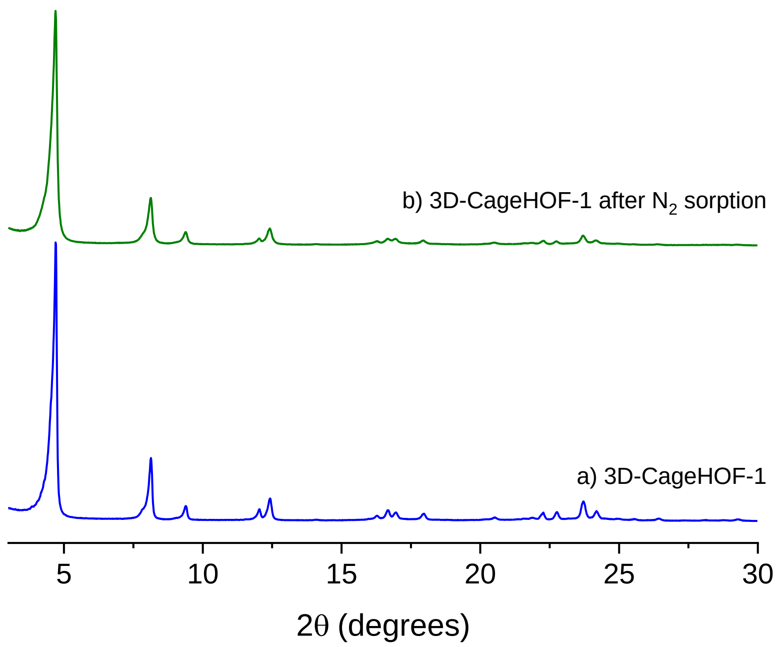

Figure S29. Comparison of PXRD patterns of **3D-CageHOF-1** recorded before (a) and after (b) N_2 sorption at 77 K.

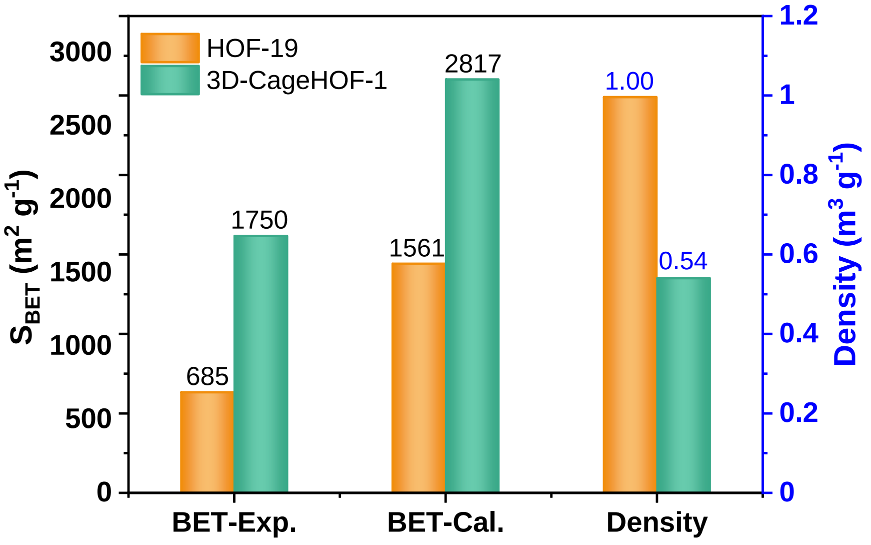

Figure S30. Comparison of experimental and simulated BET surface area and simulated density between **3D-CageHOF-1** and HOF-19. The simulated BET surface areas and densities were calculated by zeo++ using the probe size of 1.2 Å.

Section 7 Single Crystal Data for T2-ε and 3D-CageHOF-1

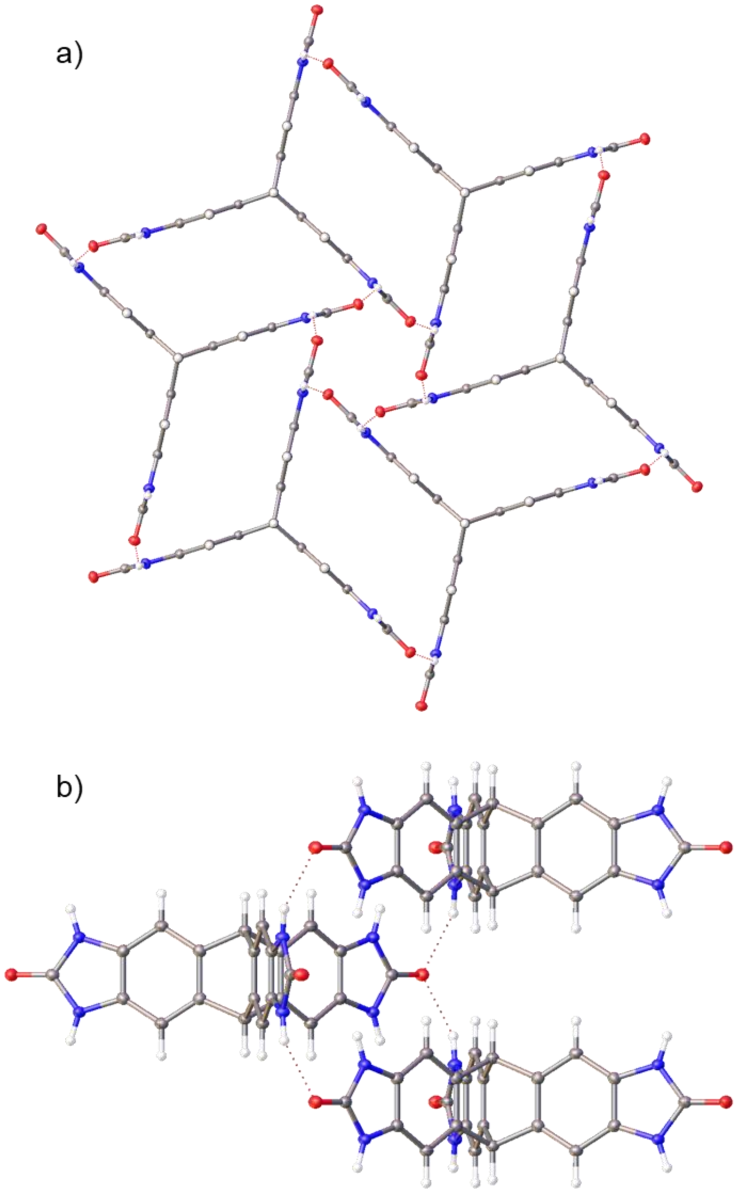

Figure S31. Displacement ellipsoid plots from the single crystal structure of **T2**-ε; two views are shown, top view (a), front view (b). C = grey; H = white; N = blue; O = red.

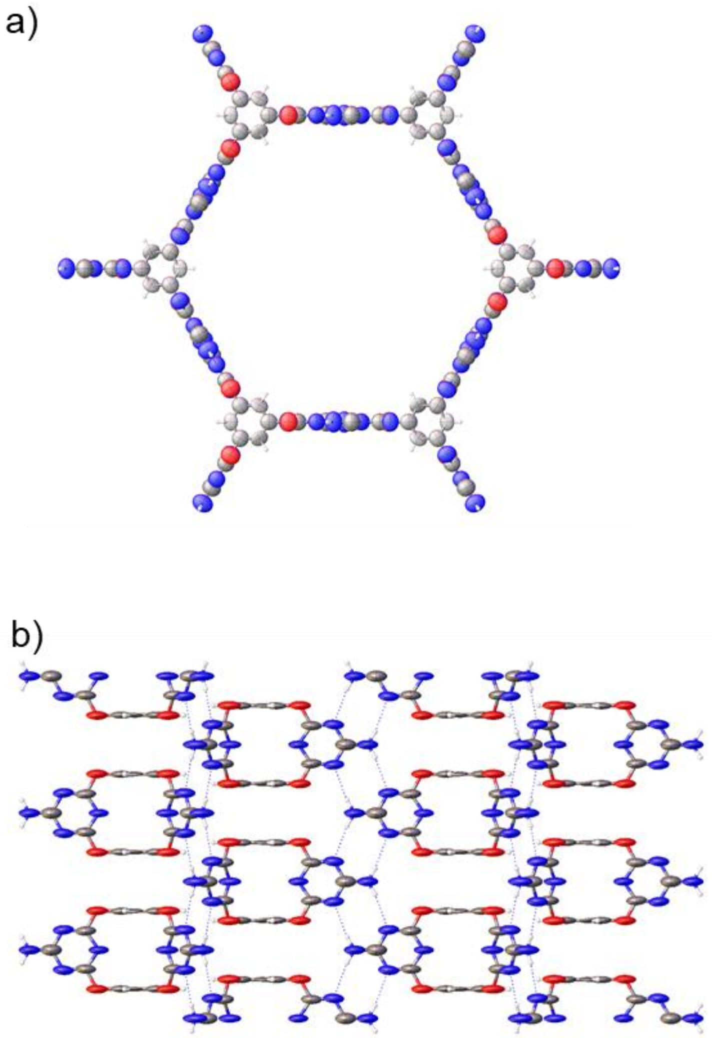

Figure S32. Displacement ellipsoid plots from the single crystal structure of **3D-CageHOF-1** ; two views are shown, top view (a), front view (b). C = grey; H = white; N = blue; O = red.

Table S7. Single crystal refinement details for **T2**-ε.

**Name**	**T2**-ε
**Crystallization solvent**	sublimed
**Wavelength/ Å**	Mo-Kα, 0.71073
**Formula**	$C_{23} H_{14} N_6 O_3$
**Weight**	422.40
**Crystal system**	hexagonal
**Space group**	$P6_3/m$
$a = b$ **(Å)**	12.6079(11)
c **(Å)**	7.4937(7)
$\alpha = \beta$ **(°)**	90
γ **(°)**	120
V **(Å^3)**	1031.6(2)
ρ_{calcd} **(g cm^{-1})**	1.360
Z	2
T **(K)**	100
μ **(mm^{-1})**	0.095
F**(000)**	436
θ **range (°)**	1.865–27.995
**Reflections collected**	7438
**Independent reflections**	892
**Obs. Data [$I > 2\sigma$]**	751
**Data / restraints / parameters**	892 / 0 / 56
R_{int}	0.0533
**Final R_1 values ($I > 2\sigma(I)$)**	0.0337
**Final R_1 values (all data)**	0.0424
wR_2 **(all data)**	0.0922
**Goodness-of-fit on $\|F\|^2$**	1.045
**CCDC**	2157173

Table S8. Single crystal refinement details for **3D-CageHOF-1**.

**Name**	**3D-CageHOF-1**
**Crystallization solvent**	Formic acid, diethyl ether, and aniline (1 molar eq per **Cage-3-NH2**)
**Wavelength/ Å**	Mo-Kα, 0.71073
**Formula**	$C_{21} H_{12} N_{12} O_6$
**Weight**	528.43
**Crystal system**	hexagonal
**Space group**	$P6_3/mmc$
$a = b$ **(Å)**	21.539(8)
c **(Å)**	7.9530(11)
$\alpha = \beta$ **(°)**	90
γ **(°)**	120
V **(Å^3)**	3195(2)
ρ **calcd (g cm^{-1})**	0.549
Z	2
T **(K)**	100
μ **(mm^{-1})**	0.043
F**(000)**	1170
θ **range (°)**	1.891–17.258
**Reflections collected**	8761
**Independent reflections**	398
**Obs. Data [$I > 2\sigma$]**	251
**Data / restraints / parameters**	398 / 60 / 43
R_{int}	0.1402
**Final R_1 values ($I > 2\sigma(I)$)**	0.0835
**Final R_1 values (all data)**	0.1357
wR_2 **(all data)**	0.2451
**Goodness-of-fit on $\|F\|^2$**	1.164
**CCDC**	2157172

References

(1) Han, B.; Wang, H.; Wang, C.; Wu, H.; Zhou, W.; Chen, B.; Jiang, J. Postsynthetic Metalation of a Robust Hydrogen-Bonded Organic Framework for Heterogeneous Catalysis. *J. Am. Chem. Soc.* **2019**, *141* (22), 8737–8740.

(2) Gaussian09, R. A.; Frisch, M. J.; Trucks, G. W.; Schlegel, H. B.; Scuseria, G. E.; Robb, M. A.; Cheeseman, J. R.; Gonzalez, J. A.; Pople, J. A. Gaussian 09, Revision E. 01, Gaussian, Inc, Wallingford, CT, 2004. *Inc., Wallingford CT* **2009**, *121*.

(3) Adamo, C.; Barone, V. Toward Reliable Density Functional Methods without Adjustable Parameters: The PBE0 Model. *J. Chem. Phys.* **1999**, *110* (13), 6158–6170.

(4) Grimme, S.; Ehrlich, S.; Goerigk, L. Effect of the Damping Function in Dispersion Corrected Density Functional Theory. *J. Comput. Chem.* **2011**, *32* (7), 1456–1465.

(5) Case, D. H.; Campbell, J. E.; Bygrave, P. J.; Day, G. M. Convergence Properties of Crystal Structure Prediction by Quasi-Random Sampling. *J. Chem. Theory Comput.* **2016**, *12* (2), 910–924.

(6) Coombes, D. S.; Price, S. L.; Willock, D. J.; Leslie, M. Role of Electrostatic Interactions in Determining the Crystal Structures of Polar Organic Molecules. A Distributed Multipole Study. *J. Phys. Chem.* **1996**, *100* (18), 7352–7360.

(7) Becke, A. D. Density-Functional Thermochemistry. III. The Role of Exact Exchange. *J. Chem. Phys.* **1993**, *98* (7), 5648–5652.

(8) Stone, A. J. Distributed Multipole Analysis: Stability for Large Basis Sets. *J. Chem. Theory Comput.* **2005**, *1* (6), 1128–1132.

(9) Ferenczy, G. G.; Winn, P. J.; Reynolds, C. A. Toward Improved Force Fields. 2. Effective Distributed Multipoles. *J. Phys. Chem. A* **1997**, *101* (30), 5446–5455.

(10) Yang, S.; Day, G. M. Exploration and Optimization in Crystal Structure Prediction: Combining Basin Hopping with Quasi-Random Sampling. *J. Chem. Theory Comput.* **2021**, *17* (3), 1988–1999.

(11) Gaus, M.; Cui, Q.; Elstner, M. DFTB3: Extension of the Self-Consistent-Charge Density-Functional Tight-Binding Method (SCC-DFTB). *J. Chem. Theory Comput.* **2011**, *7* (4), 931–948.

(12) Hourahine, B.; Aradi, B.; Blum, V.; Bonafé, F.; Buccheri, A.; Camacho, C.; Cevallos, C.; Deshaye, M. Y.; Dumitric, T.; Dominguez, A.; Ehlert, S.; Elstner, M.; Van Der Heide, T.; Hermann, J.; Irle, S.; Kranz, J. J.; Köhler, C.; Kowalczyk, T.; Kubař, T.; Lee, I. S.; Lutsker, V.; Maurer, R. J.; Min, S. K.; Mitchell, I.; Negre,

C.; Niehaus, T. A.; Niklasson, A. M. N.; Page, A. J.; Pecchia, A.; Penazzi, G.; Persson, M. P.; Å&tild;ezáč, J.; Sánchez, C. G.; Sternberg, M.; Stöhr, M.; Stuckenberg, F.; Tkatchenko, A.; Yu, V. W. Z.; Frauenheim, T. DFTB+, a Software Package for Efficient Approximate Density Functional Theory Based Atomistic Simulations. *J. Chem. Phys.* **2020**, *152* (12), 124101.

(13) Gaus, M.; Goez, A.; Elstner, M. Parametrization and Benchmark of DFTB3 for Organic Molecules. *J. Chem. Theory Comput.* **2013**, *9* (1), 338–354.

(14) Iuzzolino, L.; McCabe, P.; Price, S. L.; Brandenburg, J. G. Crystal Structure Prediction of Flexible Pharmaceutical-like Molecules: Density Functional Tight-Binding as an Intermediate Optimisation Method and for Free Energy Estimation. *Faraday Discuss.* **2018**, *211*, 275–296.

(15) Pulido, A.; Chen, L.; Kaczorowski, T.; Holden, D.; Little, M. A.; Chong, S. Y.; Slater, B. J.; McMahon, D. P.; Bonillo, B.; Stackhouse, C. J.; Stephenson, A.; Kane, C. M.; Clowes, R.; Hasell, T.; Cooper, A. I.; Day, G. M. Functional Materials Discovery Using Energy-Structure-Function Maps. *Nature* **2017**, *543* (7647), 657–664.

(16) Widdowson, D.; Mosca, M. M.; Pulido, A.; Cooper, A. I.; Kurlin, V. Average Minimum Distances of Periodic Point Sets – Foundational Invariants for Mapping Periodic Crystals. *Match* **2021**, *87* (3), 529–559.

(17) Widdowson, D.; Kurlin, V. Pointwise Distance Distributions of Periodic Sets. *arXiv*, [preprint] 2021-08-10 [accessed 2022-04-14], URL: https://doi.org/10.48550/arXiv.2108.04798.

(18) Hargreaves, C. J.; Dyer, M. S.; Gaultois, M. W.; Kurlin, V. A.; Rosseinsky, M. J. The Earth Mover's Distance as a Metric for the Space of Inorganic Compositions. *Chem. Mater.* **2020**, *32* (24), 10610–10620.

(19) Sheldrick, G. SHELXT. *Acta Cryst.* **2015**, *A71* (A71), 3–8.

(20) Sheldrick, G. SHELXL. *Acta Cryst.* **2015**, *C71* (C71), 3–8.

(21) O. V. Dolomanov R. J. Gildea, J. A. K. Howard and H. Puschmann, L. J. B. OLEX2. *J. Appl. Cryst.* **2009**, *42* (42), 339–341.

(22) Sheldrick, G. M. SADABS, Programs for Scaling and Absorption Correction of Area Detector Data. *SADABS, Programs Scaling Absorpt. Correct. Area Detect. Data* **1997**, University of Göttingen: Göttingen, Germany.

(23) Krause, L.; Herbst-Irmer, R.; Sheldrick, G. M.; Stalke, D. Comparison of Silver and Molybdenum Microfocus X-Ray Sources for Single-Crystal Structure Determination. *J. Appl. Crystallogr.* **2015**, *48* (1), 3–10.

(24) Burla, M. C.; Caliandro, R.; Camalli, M.; Carrozzini, B.; Cascarano, G. L.; De Caro, L.; Giacovazzo, C.;

Polidori, G.; Spagna, R. SIR2004: An Improved Tool for Crystal Structure Determination and Refinement. *J. Appl. Crystallogr.* **2005**, *38* (2), 381–388.

(25) Wang, D. X.; Wang, Q. Q.; Han, Y.; Wang, Y.; Huang, Z. T.; Wang, M. X. Versatile Anion-π Interactions between Halides and a Conformationally Rigid Bis(Tetraoxacalix[2]Arene[2]Triazine) Cage and Their Directing Effect on Molecular Assembly. *Chem. Eur. J.* **2010**, *16* (44), 13053–13057.

(26) He, Y.; Xiang, S.; Chen, B. A Microporous Hydrogen-Bonded Organic Framework for Highly Selective C 2H2/C2H4 Separation at Ambient Temperature. *J. Am. Chem. Soc.* **2011**, *133* (37), 14570–14573.

(27) Yang, W.; Greenaway, A.; Lin, X.; Matsuda, R.; Blake, A. J.; Wilson, C.; Lewis, W.; Hubberstey, P.; Kitagawa, S.; Champness, N. R.; Schröder, M. Exceptional Thermal Stability in a Supramolecular Organic Framework: Porosity and Gas Storage. *J. Am. Chem. Soc.* **2010**, *132* (41), 14457–14469.

(28) Mastalerz, M.; Oppel, I. M. Rational Construction of an Extrinsic Porous Molecular Crystal with an Extraordinary High Specific Surface Area. *Angew. Chem. Int. Ed.* **2012**, *51* (21), 5252–5255.

(29) Yamamoto, A.; Hirukawa, T.; Hisaki, I.; Miyata, M.; Tohnai, N. Multifunctionalized Porosity in Zeolitic Diamondoid Porous Organic Salt: Selective Adsorption and Guest-Responsive Fluorescent Properties. *Tetrahedron Lett.* **2013**, *54* (10), 1268–1273.

(30) Natarajan, R.; Bridgland, L.; Sirikulkajorn, A.; Lee, J. H.; Haddow, M. F.; Magro, G.; Ali, B.; Narayanan, S.; Strickland, P.; Charmant, J. P. H.; Orpen, A. G.; McKeown, N. B.; Bezzu, C. G.; Davis, A. P. Tunable Porous Organic Crystals: Structural Scope and Adsorption Properties of Nanoporous Steroidal Ureas. *J. Am. Chem. Soc.* **2013**, *135* (45), 16912–16925.

(31) Li, P.; He, Y.; Guang, J.; Weng, L.; Zhao, J. C. G.; Xiang, S.; Chen, B. A Homochiral Microporous Hydrogen-Bonded Organic Framework for Highly Enantioselective Separation of Secondary Alcohols. *J. Am. Chem. Soc.* **2014**, *136* (2), 547–549.

(32) Li, P.; He, Y.; Arman, H. D.; Krishna, R.; Wang, H.; Weng, L.; Chen, B. A Microporous Six-Fold Interpenetrated Hydrogen-Bonded Organic Framework for Highly Selective Separation of C_2H_4/C_2H_6. *Chem. Commun.* **2014**, *50* (86), 13081–13084.

(33) Lü, J.; Perez-Krap, C.; Suyetin, M.; Alsmail, N. H.; Yan, Y.; Yang, S.; Lewis, W.; Bichoutskaia, E.; Tang, C. C.; Blake, A. J.; Cao, R.; Schröder, M. A Robust Binary Supramolecular Organic Framework (SOF) with High CO_2 Adsorption and Selectivity. *J. Am. Chem. Soc.* **2014**, *136* (37), 12828–12831.

(34) Li, P.; He, Y.; Zhao, Y.; Weng, L.; Wang, H.; Krishna, R.; Wu, H.; Zhou, W.; O'Keeffe, M.; Han, Y.; Chen, B. A Rod-Packing Microporous Hydrogen-Bonded Organic Framework for Highly Selective Separation of

C_2H_2 /CO_2 at Room Temperature. *Angew. Chem. Int. Ed.* **2014**, *54* (2), 574–577.

(35) Wang, H.; Li, B.; Wu, H.; Hu, T. L.; Yao, Z.; Zhou, W.; Xiang, S.; Chen, B. A Flexible Microporous Hydrogen-Bonded Organic Framework for Gas Sorption and Separation. *J. Am. Chem. Soc.* **2015**, *137* (31), 9963–9970.

(36) Yang, W.; Yang, F.; Hu, T. L.; King, S. C.; Wang, H.; Wu, H.; Zhou, W.; Li, J. R.; Arman, H. D.; Chen, B. Microporous Diaminotriazine-Decorated Porphyrin-Based Hydrogen-Bonded Organic Framework: Permanent Porosity and Proton Conduction. *Cryst. Growth Des.* **2016**, *16* (10), 5831–5835.

(37) Ji, Q.; Le, H. T. M.; Wang, X.; Chen, Y. S.; Makarenko, T.; Jacobson, A. J.; Miljanic, O. Cyclotetrabenzoin: Facile Synthesis of a Shape-Persistent Molecular Square and Its Assembly into Hydrogen-Bonded Nanotubes. *Chem. Eur. J.* **2015**, *21* (48), 17205–17209.

(38) Zentner, C. A.; Lai, H. W. H.; Greenfield, J. T.; Wiscons, R. A.; Zeller, M.; Campana, C. F.; Talu, O.; Fitzgerald, S. A.; Rowsell, J. L. C. High Surface Area and Z′ in a Thermally Stable 8-Fold Polycatenated Hydrogen-Bonded Framework. *Chem. Commun.* **2015**, *51* (58), 11642–11645.

(39) Nandi, S.; Chakraborty, D.; Vaidhyanathan, R. A Permanently Porous Single Molecule H-Bonded Organic Framework for Selective CO_2 Capture. *Chem. Commun.* **2016**, *52* (45), 7249–7252.

(40) Zhou, D. D.; Xu, Y. T.; Lin, R. B.; Mo, Z. W.; Zhang, W. X.; Zhang, J. P. High-Symmetry Hydrogen-Bonded Organic Frameworks: Air Separation and Crystal-to-Crystal Structural Transformation. *Chem. Commun.* **2016**, *52* (28), 4991–4994.

(41) Hisaki, I.; Ikenaka, N.; Gomez, E.; Cohen, B.; Tohnai, N.; Douhal, A. Hexaazatriphenylene-Based Hydrogen-Bonded Organic Framework with Permanent Porosity and Single-Crystallinity. *Chem. Eur. J.* **2017**, *23* (48), 11611–11619.

(42) Hu, F.; Liu, C.; Wu, M.; Pang, J.; Jiang, F.; Yuan, D.; Hong, M. An Ultrastable and Easily Regenerated Hydrogen-Bonded Organic Molecular Framework with Permanent Porosity. *Angew. Chem. Int. Ed.* **2017**, *56* (8), 2101–2104.

(43) Wang, H.; Wu, H.; Kan, J.; Chang, G.; Yao, Z.; Li, B.; Zhou, W.; Xiang, S.; Cong-Gui Zhao, J.; Chen, B. A Microporous Hydrogen-Bonded Organic Framework with Amine Sites for Selective Recognition of Small Molecules. *J. Mater. Chem. A* **2017**, *5* (18), 8292–8296.

(44) Yan, W.; Yu, X.; Yan, T.; Wu, D.; Ning, E.; Qi, Y.; Han, Y. F.; Li, Q. A Triptycene-Based Porous Hydrogen-Bonded Organic Framework for Guest Incorporation with Tailored Fitting. *Chem. Commun.* **2017**, *53* (26), 3677–3680.

(45) Yang, W.; Wang, J.; Wang, H.; Bao, Z.; Zhao, J. C. G.; Chen, B. Highly Interpenetrated Robust Microporous Hydrogen-Bonded Organic Framework for Gas Separation. *Cryst. Growth Des.* **2017**, *17* (11), 6132–6137.

(46) Bassanetti, I.; Bracco, S.; Comotti, A.; Negroni, M.; Bezuidenhout, C.; Canossa, S.; Mazzeo, P. P.; Marchió, L.; Sozzani, P. Flexible Porous Molecular Materials Responsive to CO_2, CH_4 and Xe Stimuli. *J. Mater. Chem. A* **2018**, *6* (29), 14231–14239.

(47) Hisaki, I.; Suzuki, Y.; Gomez, E.; Cohen, B.; Tohnai, N.; Douhal, A. Docking Strategy To Construct Thermostable, Single-Crystalline, Hydrogen-Bonded Organic Framework with High Surface Area. *Angew. Chem. Int. Ed.* **2018**, *57* (39), 12650–12655.

(48) Xing, G.; Yan, T.; Das, S.; Ben, T.; Qiu, S. Synthesis of Crystalline Porous Organic Salts with High Proton Conductivity. *Angew. Chem. Int. Ed.* **2018**, *57* (19), 5345–5349.

(49) Li, P.; Li, P.; Ryder, M. R.; Liu, Z.; Stern, C. L.; Farha, O. K.; Stoddart, J. F. Interpenetration Isomerism in Triptycene-Based Hydrogen-Bonded Organic Frameworks. *Angew. Chem. Int. Ed.* **2019**, *58* (6), 1664–1669.

(50) Li, P.; Chen, Z.; Ryder, M. R.; Stern, C. L.; Guo, Q. H.; Wang, X.; Farha, O. K.; Stoddart, J. F. Assembly of a Porous Supramolecular Polyknot from Rigid Trigonal Prismatic Building Blocks. *J. Am. Chem. Soc.* **2019**, *141* (33), 12998–13002.

(51) Xing, G.; Bassanetti, I.; Bracco, S.; Negroni, M.; Bezuidenhout, C.; Ben, T.; Sozzani, P.; Comotti, A. A Double Helix of Opposite Charges to Form Channels with Unique CO_2 Selectivity and Dynamics. *Chem. Sci.* **2019**, *10* (3), 730–736.

(52) Zhou, Y.; Kan, L.; Eubank, J. F.; Li, G.; Zhang, L.; Liu, Y. Self-Assembly of Two Robust 3D Supramolecular Organic Frameworks from a Geometrically Non-Planar Molecule for High Gas Selectivity Performance. *Chem. Sci.* **2019**, *10* (26), 6565–6571.

(53) Zhang, X.; Li, L.; Wang, J. X.; Wen, H. M.; Krishna, R.; Wu, H.; Zhou, W.; Chen, Z. N.; Li, B.; Qian, G.; Chen, B. Selective Ethane/Ethylene Separation in a Robust Microporous Hydrogen-Bonded Organic Framework. *J. Am. Chem. Soc.* **2020**, *142* (1), 633–640.

(54) Zhang, X.; Wang, J. X.; Li, L.; Pei, J.; Krishna, R.; Wu, H.; Zhou, W.; Qian, G.; Chen, B.; Li, B. A Rod-Packing Hydrogen-Bonded Organic Framework with Suitable Pore Confinement for Benchmark Ethane/Ethylene Separation. *Angew. Chem. Int. Ed.* **2021**, *60* (18), 10304–10310.

(55) Li, Y. L.; Alexandrov, E. V.; Yin, Q.; Li, L.; Fang, Z. Bin; Yuan, W.; Proserpio, D. M.; Liu, T. F. Record Complexity in the Polycatenation of Three Porous Hydrogen-Bonded Organic Frameworks with Stepwise Adsorption Behaviors. *J. Am. Chem. Soc.* **2020**, *142* (15), 7218–7224.

(56) Yang, Y.; Li, L.; Lin, R. B.; Ye, Y.; Yao, Z.; Yang, L.; Xiang, F.; Chen, S.; Zhang, Z.; Xiang, S.; Chen, B. Ethylene/Ethane Separation in a Stable Hydrogen-Bonded Organic Framework through a Gating Mechanism. *Nat. Chem.* **2021**, *13* (10), 933–939.

(57) Wang, Y.; Liu, D.; Yin, J.; Shang, Y.; Du, J.; Kang, Z.; Wang, R.; Chen, Y.; Sun, D.; Jiang, J. An Ultrafast Responsive NO_2 Gas Sensor Based on a Hydrogen-Bonded Organic Framework Material. *Chem. Commun.* **2020**, *56* (5), 703–706.

(58) Feng, S.; Shang, Y.; Wang, Z.; Kang, Z.; Wang, R.; Jiang, J.; Fan, L.; Fan, W.; Liu, Z.; Kong, G.; Feng, Y.; Hu, S.; Guo, H.; Sun, D. Fabrication of a Hydrogen-Bonded Organic Framework Membrane through Solution Processing for Pressure-Regulated Gas Separation. *Angew. Chem. Int. Ed.* **2020**, *59* (10), 3840–3845.

(59) Takeda, T.; Ozawa, M.; Akutagawa, T. Jumping Crystal of a Hydrogen-Bonded Organic Framework Induced by the Collective Molecular Motion of a Twisted π System. *Angew. Chem. Int. Ed.* **2019**, *58* (30), 10345–10352.

(60) Gong, W.; Chu, D.; Jiang, H.; Chen, X.; Cui, Y.; Liu, Y. Permanent Porous Hydrogen-Bonded Frameworks with Two Types of Brønsted Acid Sites for Heterogeneous Asymmetric Catalysis. *Nat. Commun.* **2019**, *10* (1), 600.

(61) Yin, Q.; Li, Y. L.; Li, L.; Lü, J.; Liu, T. F.; Cao, R. Novel Hierarchical Meso-Microporous Hydrogen-Bonded Organic Framework for Selective Separation of Acetylene and Ethylene versus Methane. *ACS Appl. Mater. Interfaces* **2019**, *11* (19), 17823–17827.

(62) Gao, J.; Cai, Y.; Qian, X.; Liu, P.; Wu, H.; Zhou, W.; Liu, D. X.; Li, L.; Lin, R. B.; Chen, B. A Microporous Hydrogen-Bonded Organic Framework for the Efficient Capture and Purification of Propylene. *Angew. Chem. Int. Ed.* **2021**, *60* (37), 20400–20406.

(63) Wang, B.; He, R.; Xie, L. H.; Lin, Z. J.; Zhang, X.; Wang, J.; Huang, H.; Zhang, Z.; Schanze, K. S.; Zhang, J.; Xiang, S.; Chen, B. Microporous Hydrogen-Bonded Organic Framework for Highly Efficient Turn-Up Fluorescent Sensing of Aniline. *J. Am. Chem. Soc.* **2020**, *142* (28), 12478–12485.

(64) Yu, B.; Geng, S.; Wang, H.; Zhou, W.; Zhang, Z.; Chen, B.; Jiang, J. A Solid Transformation into Carboxyl Dimers Based on a Robust Hydrogen-Bonded Organic Framework for Propyne/Propylene Separation. *Angew. Chem. Int. Ed.* **2021**, *60* (49), 25942–25948.
